# Deregulated RNAs involved in sympathetic regulation of sepsis-induced acute lung injury based on whole transcriptome sequencing

**DOI:** 10.1186/s12864-022-09073-8

**Published:** 2022-12-16

**Authors:** Jia Zhang, Zhao Zhang, Xinran Nie, Yingli Liu, Yong Qi, Jing Wang

**Affiliations:** 1 Department of Respiratory and Critical Care Medicine, The First Affiliated Hospital of Zhengzhou University, Zhengzhou, 450052 Henan China; 2 Department of Respiratory and Critical Care Medicine, Henan Provincial People’s Hospital, People’s Hospital of Zhengzhou University, Zhengzhou, 450003 Henan China

**Keywords:** Sympathetic nerve, Acute lung injury, Sepsis, NF- κB, ncRNAs

## Abstract

Sympathetic nerves play essential roles in the regulation of lung inflammation, and we investigated the effect of sympathetic denervation (SD) on sepsis-induced acute lung injury (ALI) in mice. Mice were randomized to the control, SD, ALI and SD + ALI, groups. SD and ALI were established through intratracheal 6-hydroxydopamine and intraperitoneal lipopolysaccharide, respectively. Models and gene expressions levels were evaluated by HE staining, ELISA, Western blotting and RT-qPCR. RNA extraction, whole transcriptome sequencing and subsequent biostatistical analysis were performed. Sympathetic denervation in the lungs significantly attenuated lung TNF-ɑ and norepinephrine expression, alleviated sepsis-induced acute lung injury and inhibited NF-κB signaling. Compared with the ALI group, the SD + ALI group exhibited 629 DE circRNAs, 269 DE lncRNAs,7 DE miRNAs and 186 DE mRNAs, respectively. Some DE RNAs were validated by RT-qPCR. CircRNA–miRNA–mRNA regulatory networks in the SD + ALI group revealed enrichment of the B-cell receptor signaling pathway, IL-17 signaling pathway, neuroactive ligand–receptor interaction, CAM, primary immunodeficiency, and cytokine–cytokine receptor interaction terms. The lncRNA-miRNA-mRNA network also revealed inflammation–related signaling pathways. Taken together, based on the successfully established models of SD and ALI, we show here that sympathetic nerves may regulate sepsis-induced ALI supposedly by affecting the expression of circRNAs, lncRNAs, miRNAs, and mRNAs in the lungs. These results may allow for further exploration of the roles of pulmonary sympathetic nerves in sepsis-induced ALI.

**Supplementary Information:**

The online version contains supplementary material available at 10.1186/s12864-022-09073-8.

## Introduction

Acute Lung Injury (ALI) is characterized by both alveolar epithelial cell and pulmonary vascular endothelial cell injury, clinically manifested as progressively hypoxemia and dyspnea. ALI may leads to acute respiratory distress syndrome (ARDS). It is a significant public health problem that threatens human health, but effective treatment measures are lacking, and the pathogenesis is not completely clear [1]. In recent years, accumulating evidence has suggested the role of an interaction between the nervous system and immune system, but many aspects of this interaction have not been elucidated, and the roles and mechanisms of sympathetic nerves in the regulation of inflammation, especially in sepsis-induced ALI, are not completely clear.

An increasing number of non-coding RNAs (ncRNAs), including Circular RNAs (circRNAs), long noncoding RNAs (lncRNAs), and microRNAs (miRNAs), have been found in recent years. CircRNAs are a class of relatively stable ncRNAs with closed-loop structures. LncRNAs are RNAs that are longer than 200 nucleotides, while miRNAs are approximately 22 nt short-chain RNAs that can inhibit the translation of target genes or degrade target genes, thereby affecting the expression of the target genes [2]. CircRNAs or lncRNAs can competitively act on miRNA response elements, and regulate protein expression and function，and are therefore called competing endogenous RNAs (ceRNAs) [3]. CircRNAs [4–6], lncRNAs [7], and miRNAs [8, 9] play essential roles in lung injury, autoimmune disease, cancers, and other diseases, and can be used as therapeutic targets and biological markers for many diseases. Several studies have shown that sympathetic nerves can affect the level of miRNAs [10, 11]. Still, it is unclear whether sympathetic nerves affect the expression of the ncRNAs in sepsis-induced ALI.

In this study, we first established mouse sympathetic denervation (SD) models and sepsis-induced ALI models. We found that the NF-κB signaling pathway was inhibited in the SD + ALI group, and the tumor necrosis factor-α (TNF-α) level in bronchoalveolar lavage fluid (BALF) was reduced. Then, high-throughput sequencing was used to obtain the differentially expressed (DE) circRNAs, lncRNAs, miRNAs, and mRNAs in the lungs of SD mice, ALI mice, and SD + ALI mice, respectively. Based on these DE circRNAs, DE lncRNAs, DE miRNAs, and DE mRNAs, circRNA/lncRNA–miRNA–mRNA networks were constructed. Gene Ontology (GO) and Kyoto Encyclopedia of Genes and Genomes (KEGG) analyses were used to explore the possible mechanisms of these DE non-coding RNAs. RT-qPCR validated some of the meaningful ncRNAs. Collectively, our results reveal that pulmonary sympathetic nerves play an essential role in sepsis-induced acute lung disease and reveal the novel mechanisms that affect ncRNAs in sepsis-induced ALI.

## Materials and methods

### Animals

Healthy adult male C57BL/6 J mice were provided by the Experimental Animal Center (EAC) of Zhengzhou University. All mice were raised in the EAC of Zhengzhou University. A total of 40 male C57/J mice aged 6–8 weeks were randomly divided into 4 groups: the control (Ctrl) group; the SD group; the ALI group; and the SD + ALI group.

### Establishment of the SD model and sepsis-induced ALI model

To establish the SD model, we administered intratracheal (i.t.) 6-hydroxydopamine (6-OHDA, Shyuanye, Shanghai, China) by a Mice Intubation Kit (Ruiwode Inc., Shenzhen, China). Briefly, after anesthetization, the mice in the SD and SD + ALI groups were given 40 μl of i.t. 6-OHDA (10 mg/ml) for two consecutive days; the solvent of 6-OHDA was 0.01 mol/L phosphate-buffered saline + 0.1% vitamin C, and the Ctrl and ALI groups were administered with the solvent. After 10 days, the mice were subjected to the next experiment. The ALI group and the SD + ALI group were given intraperitoneal (i.p.) lipopolysaccharide (LPS, 10 mg/kg, *Escherichia coli* O55:B5, Solarbio, Beijing, China), and the Ctrl and SD groups were injected with i.p. saline. After 8 hours of saline or LPS administration, the mice were sacrificed for subsequent assays, the mortality has no difference between groups throughout the study (Fig. S1).

### Hematoxylin and eosin (HE) staining

The mice (*n* = 3) were anesthetized with isoflurane; the right lower lung tissue was fixed in 4% paraformaldehyde for 48 hours. After dehydration with graded ethanol, the tissues were embedded in paraffin, cut into 5 μm sections by a microtome, deparaffinized and rehydrated. Hematoxylin & Eosin Stain Kit (Vector Laboratories，California, USA) was used for subsequent staining according to the manufacturer’s protocol. After staining, the samples were observed under a light microscope and photographed at 200× magnification. The degree of lung injury was quantified by the lung injury score [12].

### TNF-α and norepinephrine (NE) ELISA

After the mice were anesthetized with isoflurane, blood was obtained. After standing at room temperature for 20 minutes, the blood was centrifuged for 15 minutes at room temperature at a centrifugal force of 3000 rpm in a microcentrifuge. The serum supernatant was stored at − 80 °C for subsequent analysis. To obtain BALF, the mouse neck was dissected after anesthetized, and the trachea was exposed for intubation. Three milliliters of saline was drawn and pushed into the lungs from the endotracheal tube (1 ml each time). The chest was gently squeezed, and the lavage fluid was withdrawn and the amount of recovered liquid was approximately 0.8 ml every time. The BALF was centrifuged for 15 min at 1500 rpm at 4 °C, and the supernatant was stored at − 80 °C. The TNF-α levels in serum (*n* = 4–6) or BALF (*n* = 4–6) were measured with a TNF-α ELISA kit (Abbkine, Beijing, China) according to the manufacturer’s protocols.

The right lung was collected and placed in 1 ml of NE lysis buffer (0.01 N HCl containing 4 mM sodium metabisulfite and 1 mM EDTA) and then vortexed with magnetic beads (Beads Mill 24, Fisherbrand, USA) for 20 seconds. Then, the samples were centrifuged (14,000 rpm) for 30 mins at 4 °C. The supernatant was stored at − 80 °C. An NE ELISA Kit (Abnova, Taiwan, China) was used to measure the NE level in lung tissue (*n* = 6–7) according to the manufacturer’s specifications. The protein level in the samples was used to make corrections.

### Western blot analysis

Fresh right lower lung tissue (*n* = 5–8) was collected and rapidly homogenized. Briefly, approximately 100 mg of lung tissue per mouse was added to a tube containing 500 ml of cold RIPA lysis buffer (EpiZyme, Shanghai, China) and vortexed with a machine (Beads Mill 24, Fisherbrand, USA) for 20s. The tubes were then centrifuged at 14,000 rpm for 30 minutes at 4 °C. The supernatant was aspirated and stored at − 80 °C. The protein concentration was measured with BCA regent (Solarbio, Beijing, China), and 20 μg of protein per sample was loaded into a 10% SDS–PAGE gel. Electrophoresis was performed for 1.5 h at 100 V, and the proteins were then transferred to PVDF membranes at 100 V for 1 hour. Some of the PVDF membranes were cut prior to hybridisation with antibodies. The PVDF membranes were blocked in blocking buffer (0.1% Tween 20 and 5% fetal bovine serum in 1 × PBS) for 1 hour and incubated with a primary antibody (1:200) at 4 °C overnight. After being washed 3 times with PBS-T (0.1% Tween 20 in 1× PBS), the cells were incubated with the corresponding secondary antibody (1:1000) for 1 hour at room temperature. Each membrane was washed 3 times with PBS-T, after applied ECL western HRP substrate, scanned with Highly Sensitive Chemiluminescence Imaging System (BIO-RAD, USA), and the signal values were quantified by ImageJ software.

The primary antibodies included antibodies against β-Actin (R&D Systems, Minnesota, USA), NF-κB p65 (Ser 536,Invitrogen, California, USA), NF-κB p-p65 (Ser536, Invitrogen, California, USA), IκBα (Cell Signaling Technology, Danvers, USA), p-IκBα (Ser32， Cell Signaling Technology, Danvers, USA), and tyrosine hydroxylase (TH) (Thermo Fisher, USA). The secondary antibodies included an HRP-labeled Goat Anti-Mouse IgG Secondary Antibody (Epizyme, Shanghai, China) and an HRP-labeled Goat Anti-Rabbit IgG Secondary Antibody (Epizyme, Shanghai, China).

### RNA extraction; library construction; sequencing; and identification of DE circRNAs, DE lncRNAs, and DE mRNAs

The mice (*n* = 3) were anesthetized, and lung tissues were quickly transferred to liquid nitrogen and transported to the sequencing laboratory with dry ice. The mirVana™ miRNA Isolation Kit and Ambion-1561 were used to extract total RNA, after which TruSeq Stranded Total RNA with Ribo-Zero Gold was used for library construction. Generally, the RNA was broken into short fragments. The first and second cDNA strands were synthesized in turn. dTTP was replaced with dUTP in the second cDNA strand after end repair, ligation of the sequencing adapters and digestion by the UNG enzyme. PCR amplification was then performed. After quality inspection, an Illumina sequencer was used for sequencing, and clean reads were obtained for subsequent analysis.

Briefly, the RseQC(2.6.4), fastqc(v0.11.5), HISAT2(2.2.1.0), and Stringtie2(1.3.3b) software programs were used for quality control, genome alignment and transcript splicing. The circRNAs were predicted using CIRI (v2.0.3) software [13], and the candidate lncRNAs were identified with CPC2 (beta), CNCI (1.0), PLEK (1.2) and PFAM (v30) software. The lncRNAs were compared with the Rfam databases and annotated [14]. Finally, the DE circRNAs, DE lncRNAs, and DE mRNAs were analyzed with DESeq2 (1.18.0) software. A fold change ≥2 or ≤ 0.5 and a *p* value < 0.05 were used as the cutoff criteria.

### Quantification of miRNAs and identification of DE miRNAs

After total RNA was extracted from the samples, a miRNA sequencing library was constructed using the mirVana miRNA Isolation Kit (Ambion) and TruSeq Small RNA Sample Prep Kits. Total RNA, concentration and integrity were measured by Nanodrop 2000 and Agilent 2100. After PCR amplification and quality inspection, the library was sequenced using an Illumina HiSeq X Ten platform. High-quality clean reads were obtained after filtering using Fastx-toolkit (0.0.13) software, Bowtie (1.1.1) and NGSQCToolkit.

The clean reads were aligned to the mouse genome and compared with miRBase (version 22.0). Target genes of DE miRNAs were predicted by Miranda (3.3a). The expression levels of the identified known mature miRNA sequences and the newly predicted miRNAs were quantified as the transcripts per million (TPM). DE miRNAs were analyzed with DESeq2 (1.18.0) software. The *p* values were calculated with the Audic–Claverie statistic. A fold change ≥2 or ≤ 0.5 and a *p* value < 0.05 were used as the cutoff criteria.

### Functional analysis of DE mRNAs, circRNAs, lncRNAs, and miRNAs

To understand the function of DE RNAs well, we performed GO enrichment analysis (http://geneontology.org/) and KEGG pathway analyses (http://www.genome.jp/kegg/) on DE mRNAs, GO enrichment analysis included biological process (BP), molecular function (MF), and cellular component (CC). Similar to the case for mRNAs, after the DE circRNAs, DE lncRNAs or miRNAs were obtained, GO and KEGG enrichment analyses were performed according to the circRNAs, lncRNAs or miRNAs’ predicted target genes. and a hypergeometric distribution was used to test the significance of each GO or KEGG term. The *p* value was corrected with Benjamini & Hochberg’s multiple tests to obtain the false discovery rate (FDR). Both GO terms and KEGG pathways were considered to be significantly enriched with FDR < 0.05.

### CeRNA network interaction and analysis

CircRNAs or lncRNAs regulate mRNA expression and degradation by competing with certain miRNAs, so-called ceRNAs. According to the principle of ceRNA action, the miRanda (v3.3a) program screened the circRNA/lncRNA–miRNA negative regulation relationship pairs to obtain the circRNA/lncRNA–miRNA relationship pairs. The miRanda (v3.3a) program was used to predict the relationships between miRNAs and mRNAs, and negative miRNA–mRNA relationship pairs were obtained. For these predicted relationship pairs with regulatory relationships, the MuTaME method was used to calculate the ceRNA score, which was combined with the hypergeometric distribution to calculate the *p* value of the corresponding ceRNA relationship. Finally, ceRNA relationship pairs with high reliability were obtained and used to build a ceRNA network. GO and KEGG analyses were performed based on the mRNAs in the constructed ceRNA relationship pairs.

### Real-time quantitative polymerase chain reaction (RT-qPCR) analysis

We performed RT-qPCR as we have mentioned in a previous article [15]. Relevant primers purchased from Gene–Pharma，China. Briefly, mouse lung tissue was digested, and then total RNA was extracted. After the RNA concentration was measured with a NANO 2000 Ultraviolet Spectrophotometer, the RNAs were reverse-transcribed into cDNA using β-Actin as a control. The target primers were added, and PCR was performed with a predetermined program on an Exicycler™ 96 fluorescence quantifier (Bioneer, Korea). The obtained data were further analyzed via the comparative Ct (2-ΔΔCt) method. The primers used are listed in Additional file 1.

### Statistical methods

We used One-way analysis of variance (ANOVA) tests to analyze differences between more than two groups. GraphPad Prism software 8.0 (Graph Pad Inc., La Jolla, CA) was used to analyze and graph the data.

## Results

### 6-OHDA denervates the sympathetic nerves in the lungs

**Fig. 1:**
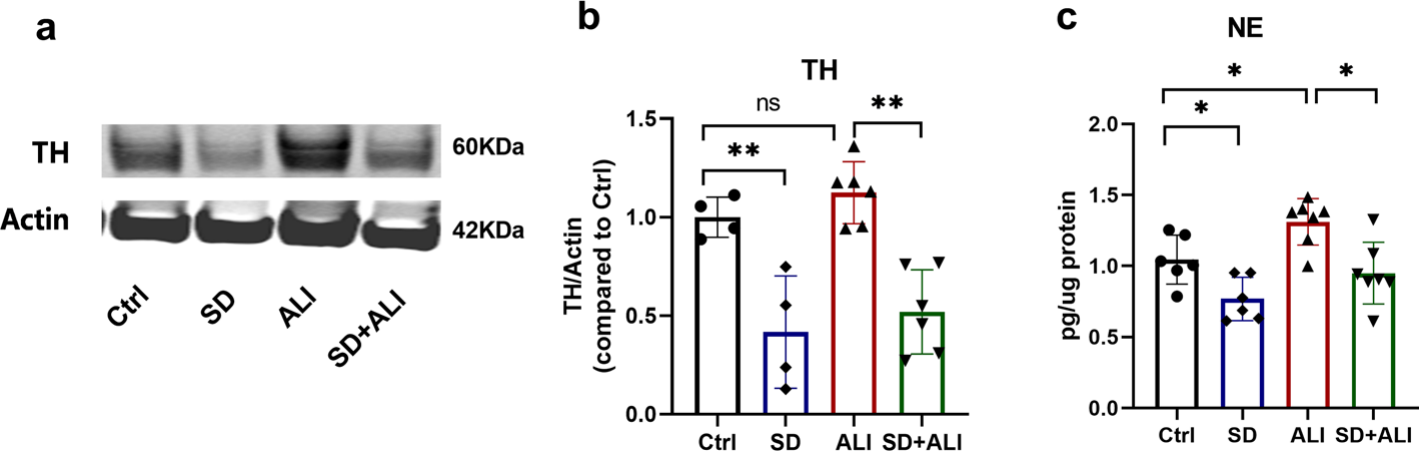
6-OHDA decreased the TH expression and NE level in the SD group. **a** expression of TH protein in 4 groups of mice lung using western blot(*n* = 4–7); **b** The bands were semi-quantitatively analyzed using the ImageJ software and normalized to β-actin; **c** NE levels in 4 groups using NE ELISA Kit(*n* = 6–7). Data represent the mean ± sd. *p < 0.05; **p < 0.01; ***p < 0.001; NS, no statistically significant difference. The Samples were derived from the same experiment and the gels/blots were processed in parallel. All of the full-length blots/gels or membrane edges visible blots are presented in Additional file 2

To validate that 6-OHDA denervates the sympathetic nerve in the lungs, we performed western blotting; the results showed that the expression of the TH protein in the SD group was significantly reduced (Fig. 1a, b), as was the NE concentration(Fig. 1c). Compared to the Ctrl group, the ALI group had higher levels of TH and NE in the lungs，although the difference in TH was not statistically significant (Fig. 1). The levels of TH and NE concentration were both lower in the SD+ ALI group than in the ALI group (Fig. 1).

### Pulmonary SD alleviates sepsis-induced ALI by inhibiting the NF-κB signaling pathway

**Fig. 2:**
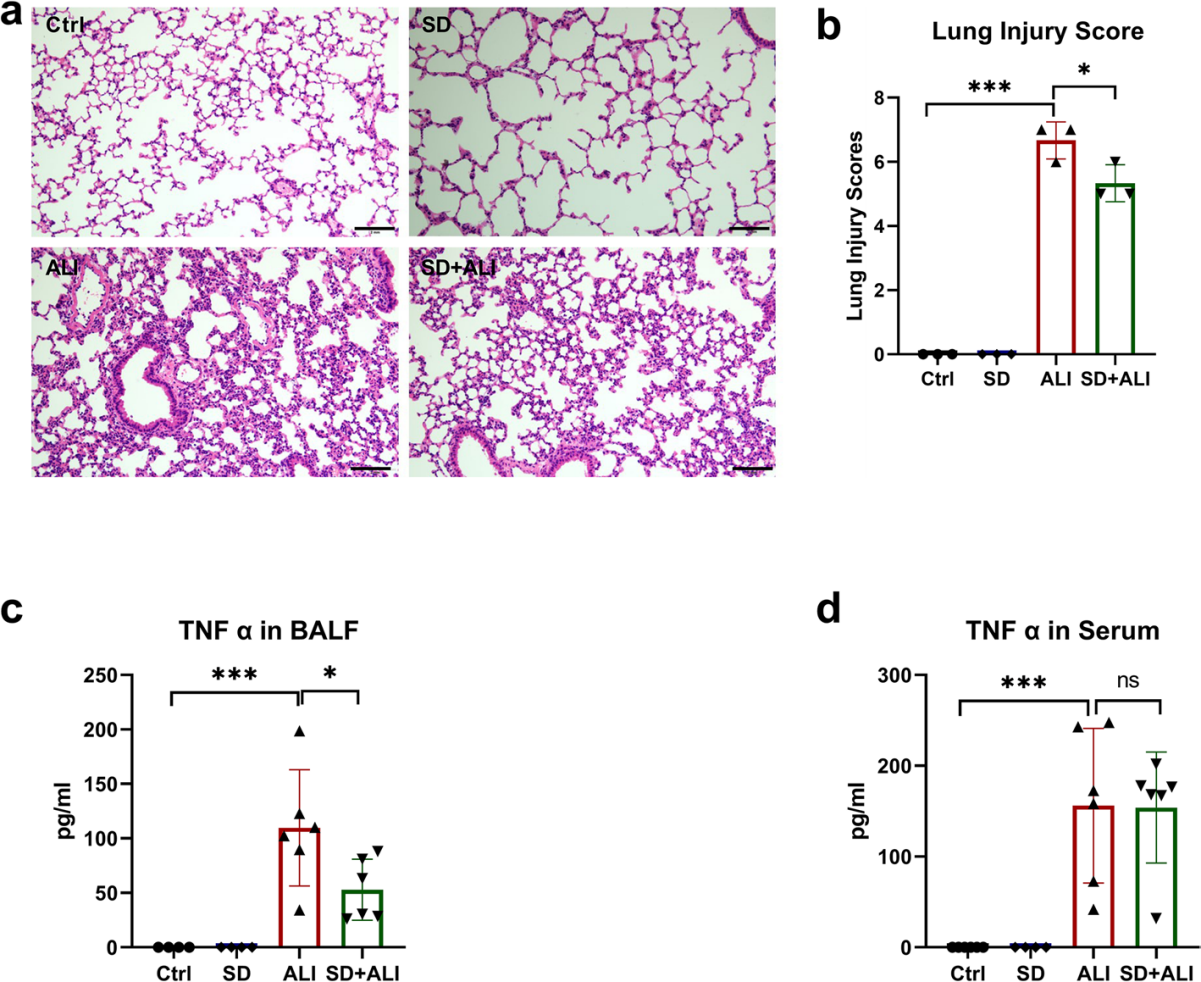
SD alleviated ALI. **a** HE staining in 4 groups; **b** lung injury score of HE staining in 4 groups (*n* = 3). **c** TNF-α levels in BALF of the 4 groups (*n* = 4–6); **d** TNF-α levels in serum of the 4 groups. Data represent the mean ± sd. *p < 0.05; **p < 0.01; ***p < 0.001; NS, no statistically significant difference. Original HE microscopy image showed in the Additional file 2

**Fig. 3:**
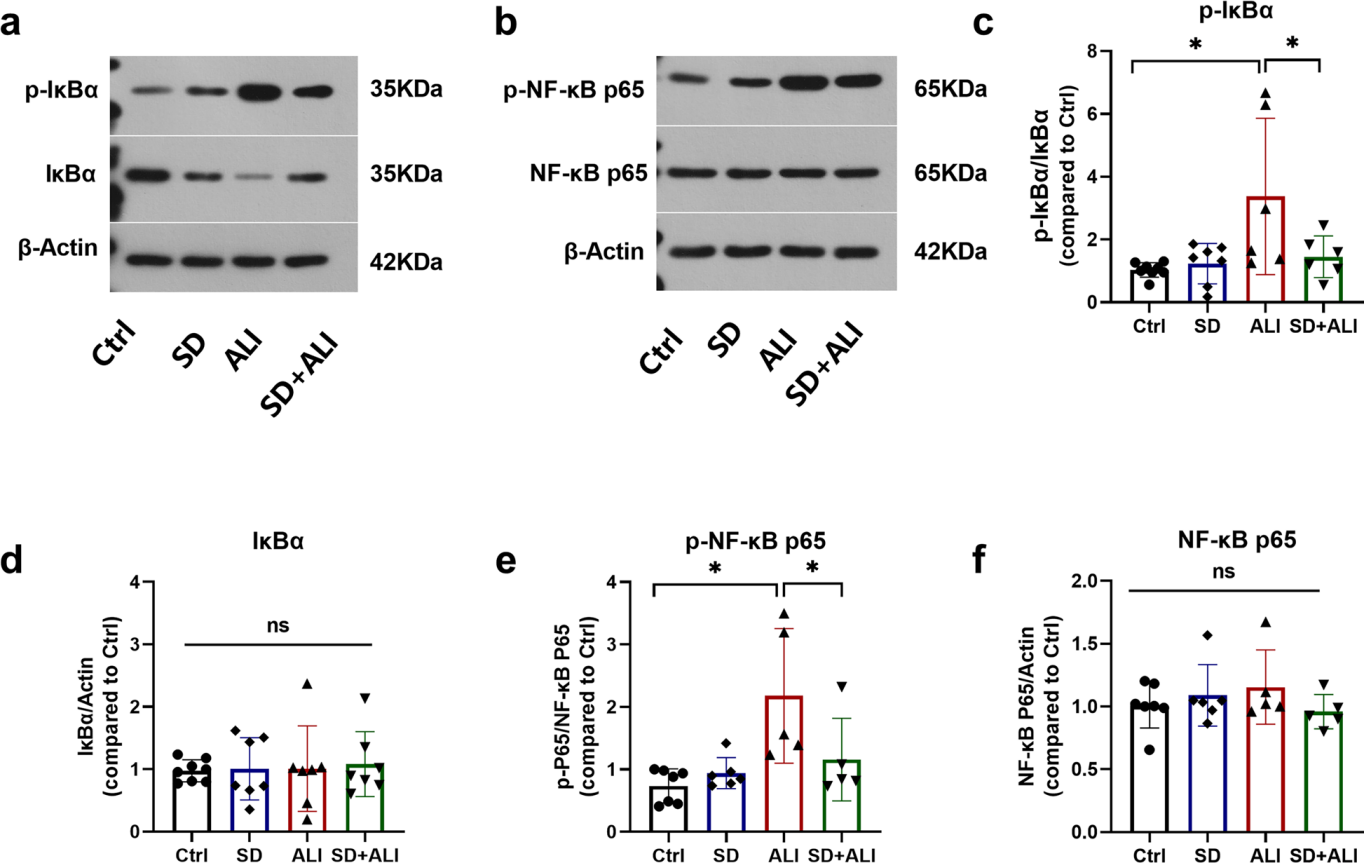
SD inhibiting ALI mice lung NF-κB signaling. **a** Sample western blot of p-IκBα, IκBα, and β-Actin proteins in 4 groups of mice lung(*n* = 5–8); **b** Sample western blot of p- NF-κB p65, NF-κB p65 and β-Actin proteins in 4 groups of mice lung(*n* = 5–8); Groups protein level data analysis of p-IκBα (**c**), IκBα (**d**), p-NF-κB p65 (**e**), NF-κB p65 (**f**), bands were semi-quantitatively analyzed by using the ImageJ software and normalized to β-actin. Data represent the mean ± sd. *p < 0.05; **p < 0.01; ***p < 0.001; NS, no statistically significant difference. The samples were derived from the same experiment and the gels/blots were processed in parallel. All of the full-length blots/gels or membrane edges visible blots are presented in Additional file 2

There was no significant difference in the lung injury score of lung tissue between the Ctrl group and the SD group (Fig. 2a, b). After LPS treatment, the lung injury score and TNF-α level in both BALF and serum were significantly increased in the ALI group (Fig. 2). SD + ALI group mice had lower lung injury scores than ALI group mice, and the levels of TNF-α in BALF, but not in serum, were consistent with the lung injury scores indicated by HE staining (Fig. 2). Correspondingly, there was no significant difference in the expression of proteins related to the NF-κB signaling pathway between the Ctrl group and SD group (Fig. 3). The expressions levels of p-IκBα (Fig. 3a, c) and p-NF-κB p65 (Fig. 3b, e) were increased in the ALI group compared with the Ctrl group, which indicated that the NF-κB signaling pathway was activated. In contrast, the NF-κB signaling pathway was inhibited in the SD + ALI group compared with the ALI group, as the expressions levels of p-IκBα (Fig. 3a, c) and p-NF-κB p65 (Fig. 3b, e) were decreased.

### DE circRNA, lncRNAs, miRNAs, and mRNAs

**Fig. 4:**
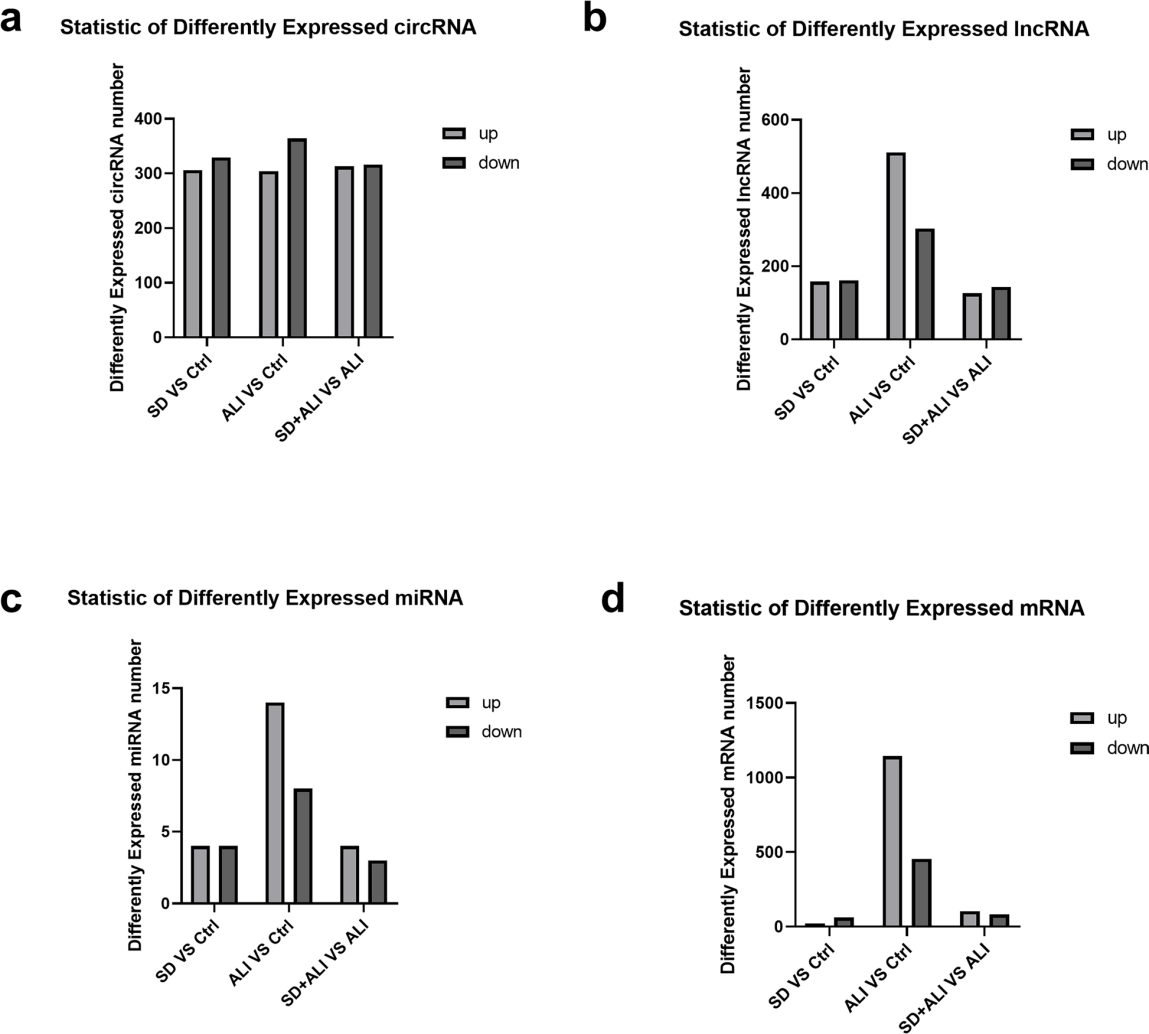
Statistic of differently expressed ncRNAs and mRNAs. **a** DE circRNAs number of the SD group compared with the Ctrl group (SD VS Ctrl), the ALI group compared with the Ctrl group (ALI VS Ctrl), the SD + ALI group compared with the ALI group (SD + ALI VS ALI); **b** DE lncRNAs number of the SD group compared with the Ctrl group (SD VS Ctrl), the ALI group compared with the Ctrl group (ALI VS Ctrl), the SD + ALI group compared with the ALI group (SD + ALI VS ALI); **c** DE miRNAs number of the SD group compared with the Ctrl group (SD VS Ctrl), the ALI group compared with the Ctrl group (ALI VS Ctrl), the SD + ALI group compared with the ALI group (SD + ALI VS ALI); **d** DE mRNAs number of the SD group compared with the Ctrl group (SD VS Ctrl), the ALI group compared with the Ctrl group (ALI VS Ctrl), the SD + ALI group compared with the ALI group (SD + ALI VS ALI)

**Fig. 5:**
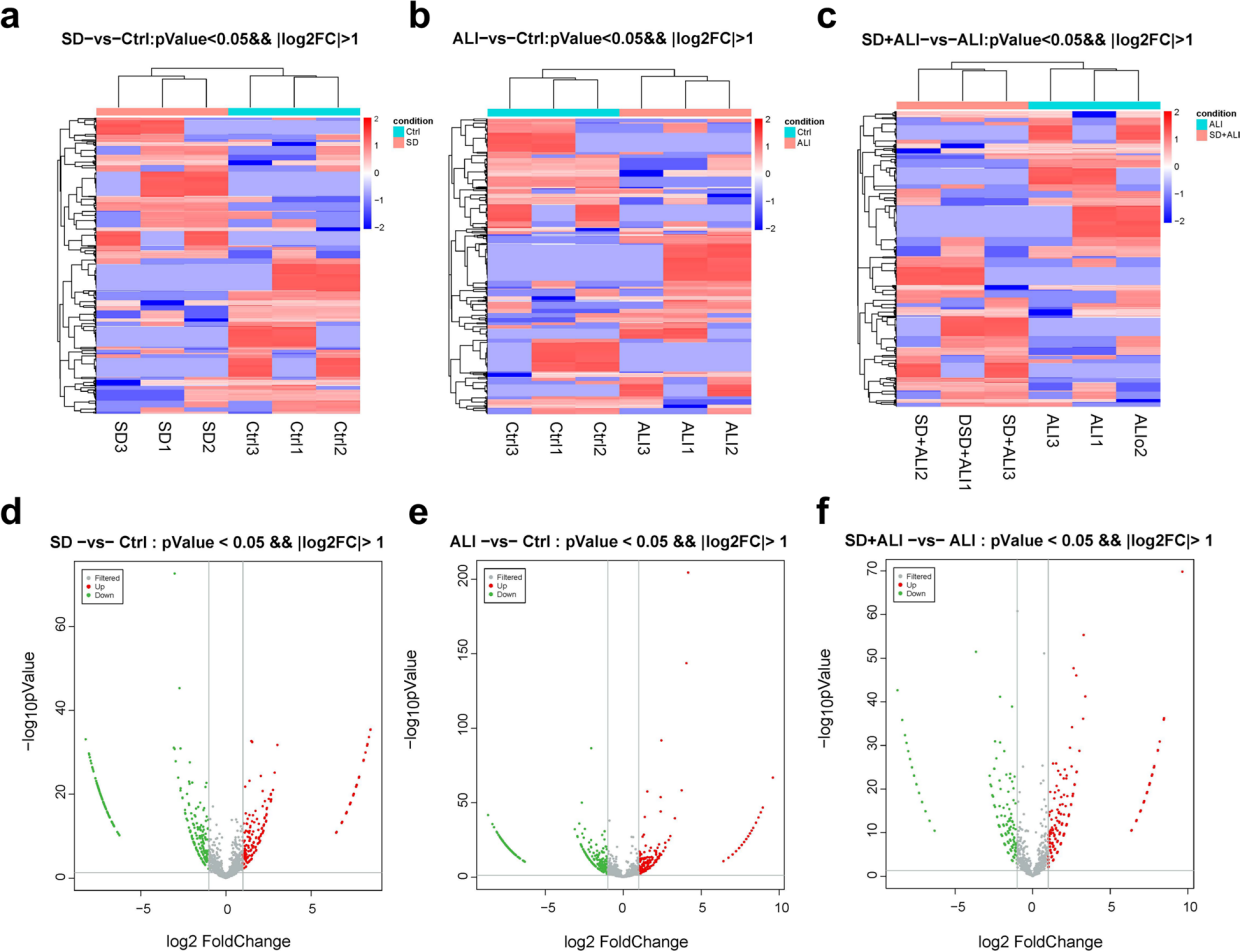
The distinct circRNAs expression profiles between groups. **a** The heat map represents hierarchical clustering for DE circRNAs in the SD group compared to the Ctrl group; **b** The heat map represents hierarchical clustering for DE circRNAs in the ALI group compared with the Ctrl group; **c** The heat map represents hierarchical clustering for DE circRNAs in the SD + ALI group compared with the ALI group. **d** The volcano plots for DE circRNAs in the SD compared with Ctrl groups; **e** The volcano plots for DE circRNAs in the ALI group compared with the Ctrl group; **f** The volcano plots for DE circRNAs in the SD + ALI group compared with the ALI group. Up-regulated expression was indicated as “red”, and down-regulated expression was indicated as “green”

**Fig. 6:**
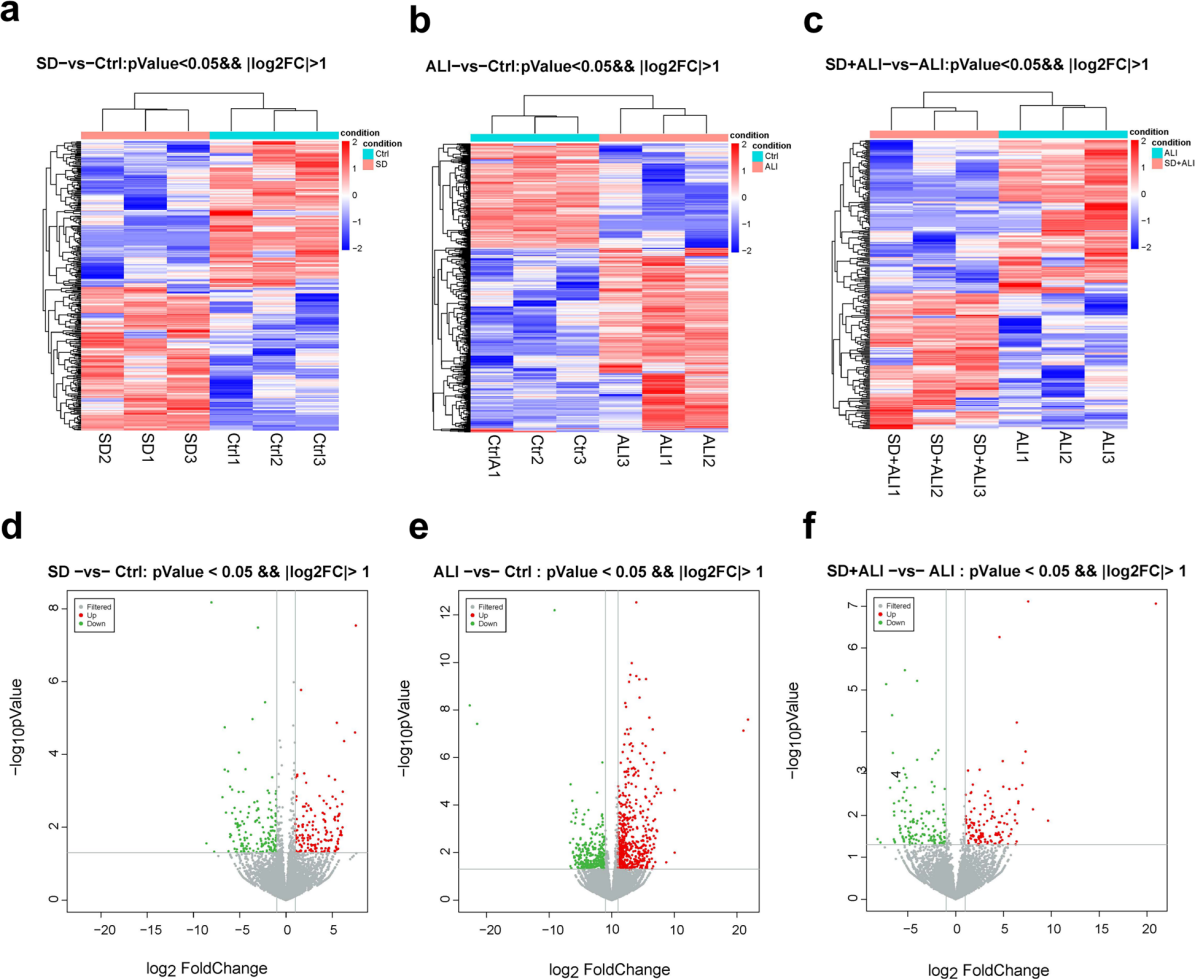
The distinct lncRNAs expression profiles between groups. **a** The heat map represents hierarchical clustering for DE lncRNAs between the SD group and Ctrl group; **b** The heat map represents hierarchical clustering for DE lncRNAs between the ALI group compared with the Ctrl group; **c** The heat map represents hierarchical clustering for DE lncRNAs in the SD + ALI group compared with the ALI group. **d** The volcano plots for DE lncRNAs in the SD compared with the Ctrl group; **e** The volcano plots for DE lncRNAs in the ALI group compared with the Ctrl group; **f** The volcano plots for DE lncRNAs in the SD + ALI group compared with the ALI group. Up-regulated expression was indicated as “red”, and down-regulated expression was indicated as “green”

**Fig. 7:**
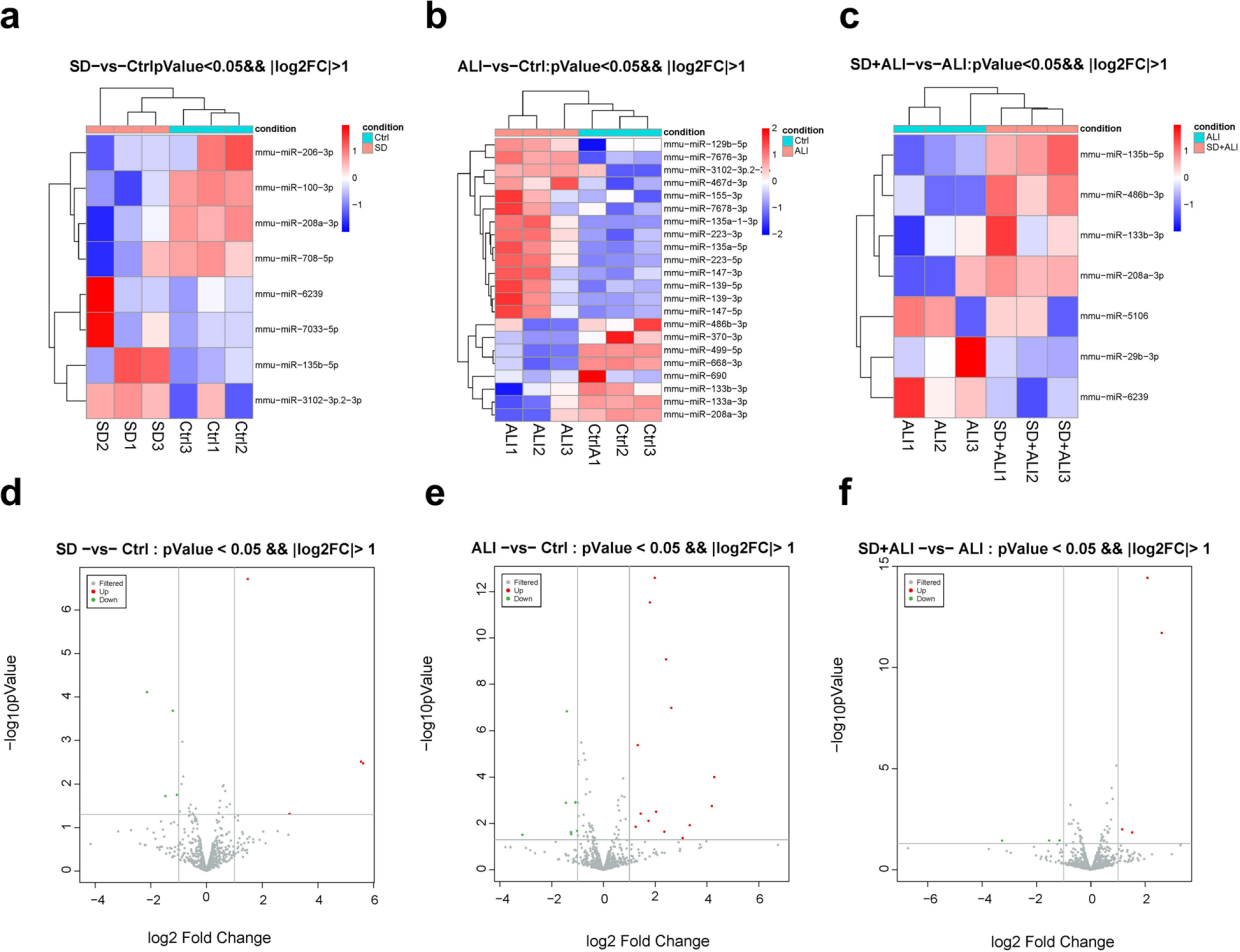
The distinct miRNAs expression profiles between groups. **a** The heat map represents hierarchical clustering for DE miRNAs between the SD group and Ctrl group; **b** The heat map represents hierarchical clustering for DE miRNAs in the ALI group compared with the Ctrl group; **c** The heat map represents hierarchical clustering for DE miRNAs in the SD + ALI group compared with the ALI group. **d** The volcano plots for DE miRNAs in the SD group compared with Ctrl group; **e** The volcano plots for DE miRNAs in the ALI group compared with the Ctrl group; **f** The volcano plots for DE miRNAs in the SD + ALI group compared with the ALI group. Up-regulated expression was indicated as “red”, and down-regulated expression was indicated as “green”

Compared with the Ctrl group, the SD group obtained fewer ncRNAs and mRNAs (Figs. 4, 5a, 5d, 6a, 6d, 7a, 7d, S2a, S2d), while compared with the Ctrl group, the ALI group exhibited 668 DE circRNAs, of which 304 were upregulated and 364 were downregulated (Figs. 4a, 5b, 5e); 814 DE lncRNAs, of which 511 were upregulated and 303 were downregulated (Figs. 4b, 6b, 6e); 22 DE miRNAs, of which 14 were upregulated and 8 were downregulated (Figs. 4c, 7b, 7e); and 1598 DE mRNAs were identified, of which 1144 were upregulated and 454 were downregulated(Fig. 4d, S2b, S2e).

Compared with the ALI group, the SD + ALI group exhibited 629 DE circRNAs, of which 313 were upregulated, and 316 were downregulated (Figs. 4a, 5c, 5f); 269 DE lncRNAs, of which 126 were upregulated, and 143 were downregulated (Figs. 4b, 6c, 6f); 7 DE miRNAs, of which 4 were upregulated and 3 were downregulated (Figs. 4c, 7c, 7f); and 186 DE mRNAs, of which 104 were upregulated and 82 were downregulated (Fig. 4d, S2c, S2f).

### Functional analysis of circRNAs, lncRNAs, miRNAs, and DE mRNAs

We performed GO and KEGG analyses on the DE circRNAs, DE lncRNAs, DE miRNAs, and DE mRNAs between groups, including the SD compared with the Ctrl group, the ALI group compared with the Ctrl group, and the SD + ALI group compared with the ALI group. The result showed that these DE RNAs in the SD group predicted few messages related to inflammation (data not shown). Meanwhile, most RNAs were predicted to relate to inflammation (Fig. S3, S4, S5, S6) in the ALI group compared with the Ctrl group, and some researchers have already reported [16, 17]. To further explore the mechanism of action of sympathetic nerves in ALI, we only focus on the result between the SD + ALI group and the ALI group as follows.

**Fig. 8:**
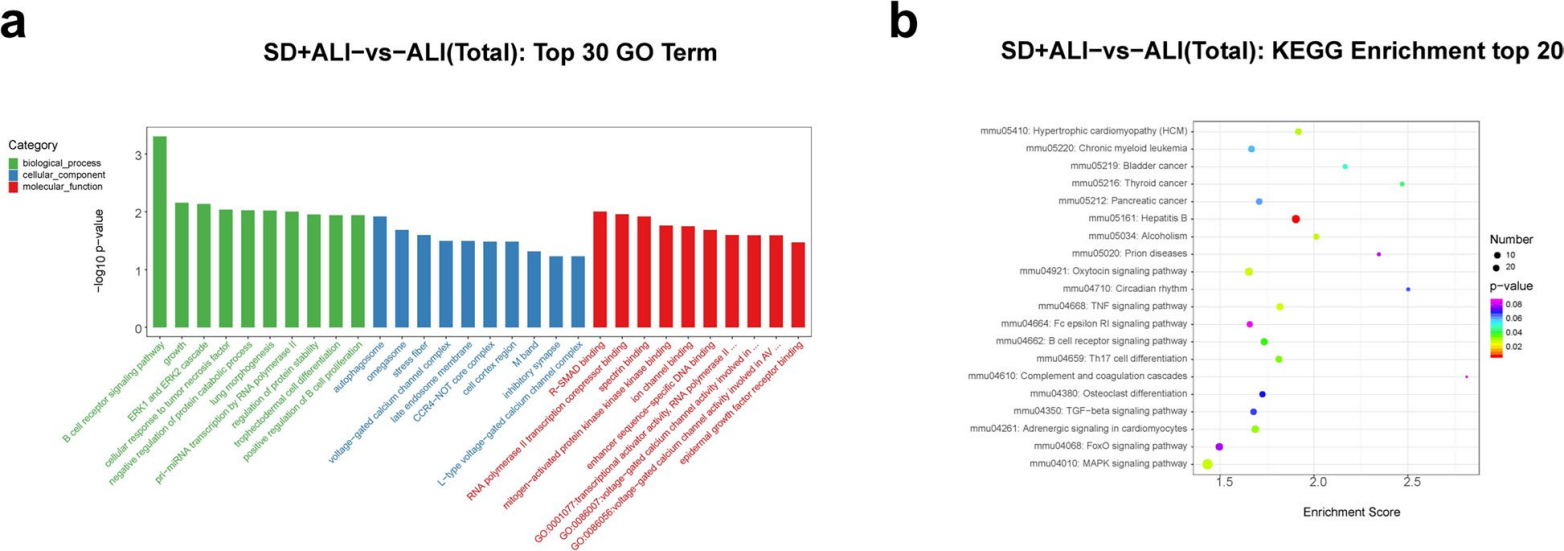
GO and KEGG analyses for the total dysregulated circRNAs in the SD + ALI group compared with the ALI group. **a** Top 30 enriched GO terms of the total dysregulated circRNAs were presented according to biological process (BP), cellular component (CC), and molecular function (MF). **b** Top 20 enriched KEGG pathways of the total dysregulated circRNAs

GO functional analysis of the DE circRNAs in the SD + ALI group compared with the ALI group indicated that the most significantly enriched BPs incuded the B-cell receptor signaling pathway, growth, extracellular regulated protein kinases (ERK) 1 and ERK2 cascade, and cellular response to tumor necrosis factor. The main CC terms included autophagosome, omegasome, and stress fiber. The main MF terms included R-SMAD binding/RNA polymerase II transcription corepressor binding and spectrin binding (Fig. 8a). The enriched KEGG pathways included the TNF signaling pathway, the oxytocin signaling pathway, the MAPK signaling pathway, Th17 cell differentiation, the B- cell receptor signaling pathway, and the TGF- β signaling pathway (Fig. 8b).

**Fig. 9:**
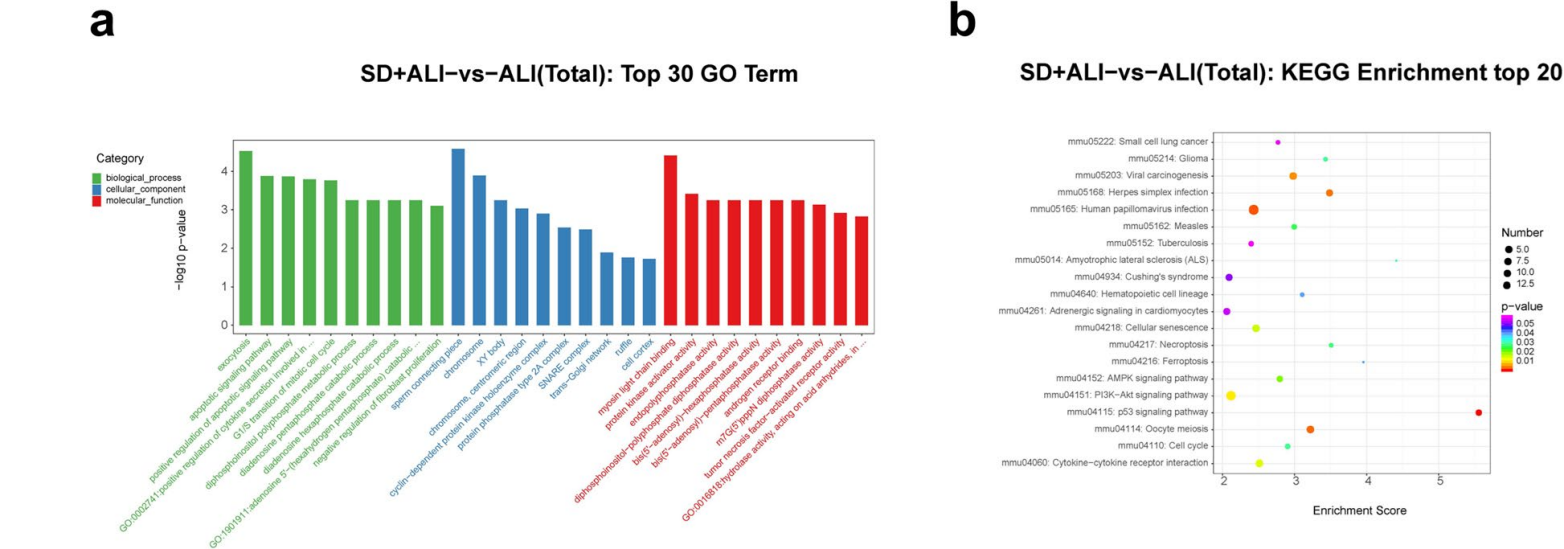
GO and KEGG analyses for the total dysregulated lncRNAs in the SD + ALI group compared with the ALI group. **a** Top 30 enriched GO terms of the total dysregulated lncRNAs were presented according to biological process (BP), cellular component (CC), and molecular function (MF). **b** Top 20 enriched KEGG pathways of the total dysregulated lncRNAs

The DE lncRNAs in the SD + ALI group were mainly associated with BP terms such as exocytosis, the apoptotic signaling pathway, and positive regulation of the apoptotic signaling pathway. The main CC terms included sperm connecting piece, chromosome, and XY body. The main MF terms included myosin light chain binding, protein kinase activity and endopolyphosphatase activity (Fig. 9a). The enriched KEGG pathways included the p53 signaling pathway, human papillomavirus infection, PI3K-Akt signaling pathway, and AMPK signaling pathway, etc. (Fig. 9b).

**Fig. 10:**
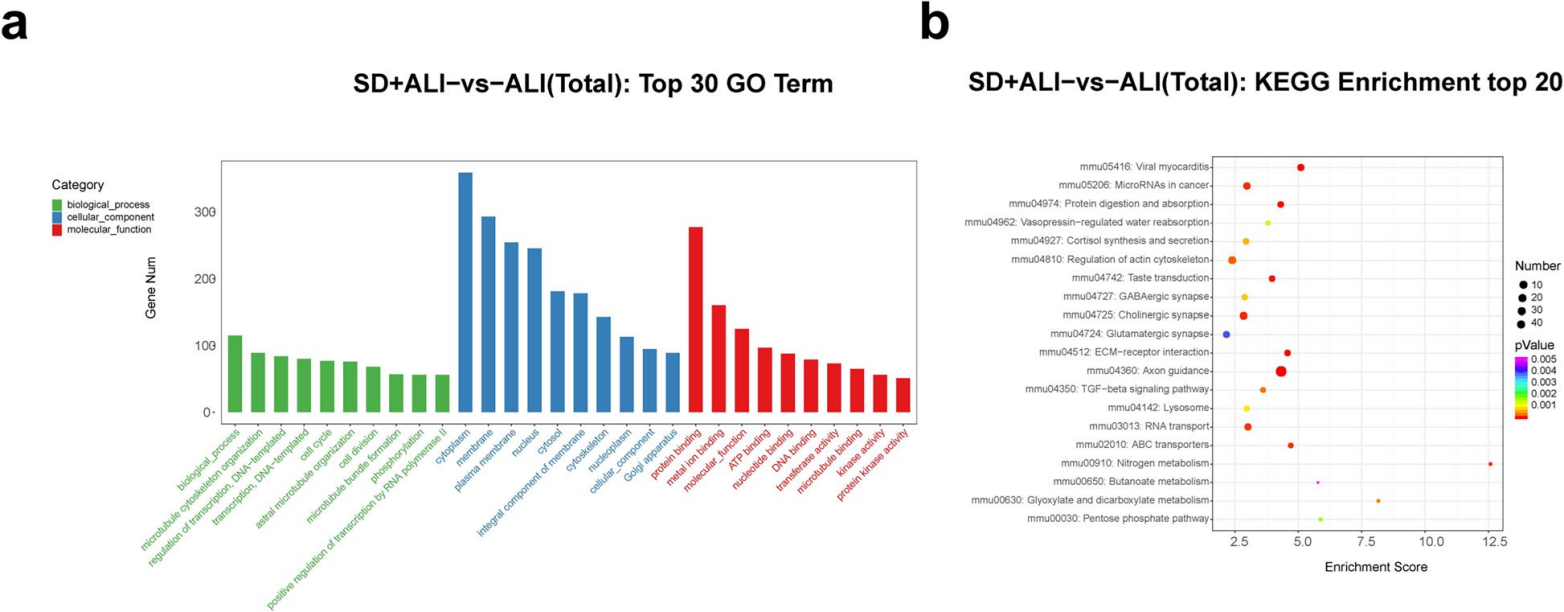
GO and KEGG analyses for the total dysregulated miRNAs in the SD + ALI group compared with the ALI group. **a** Top 30 enriched GO terms of the total dysregulated miRNAs were presented according to biological process (BP), cellular component (CC), and molecular function (MF). **b** Top 20 enriched KEGG pathways of the total dysregulated miRNAs

GO functional analysis of the DE miRNAs in the SD + ALI group revealed enrichment for the BP terms were biological process, microtubule cytoskeleton organization, and regulation of transcription; the main CC terms included cytoplasm, membrane, plasma membrane, and nucleus. The main MF terms included protein binding, metal ion binding, and molecular function, etc. (Fig. 10a), and the enriched KEGG pathways included axon guidance, viral myocarditis, Extracellular matrix (ECM)–receptor interaction, protein digestion, absorption, and the Transforming growth factor-β (TGF-β) signaling pathway, etc. (Fig. 10b).

**Fig. 11:**
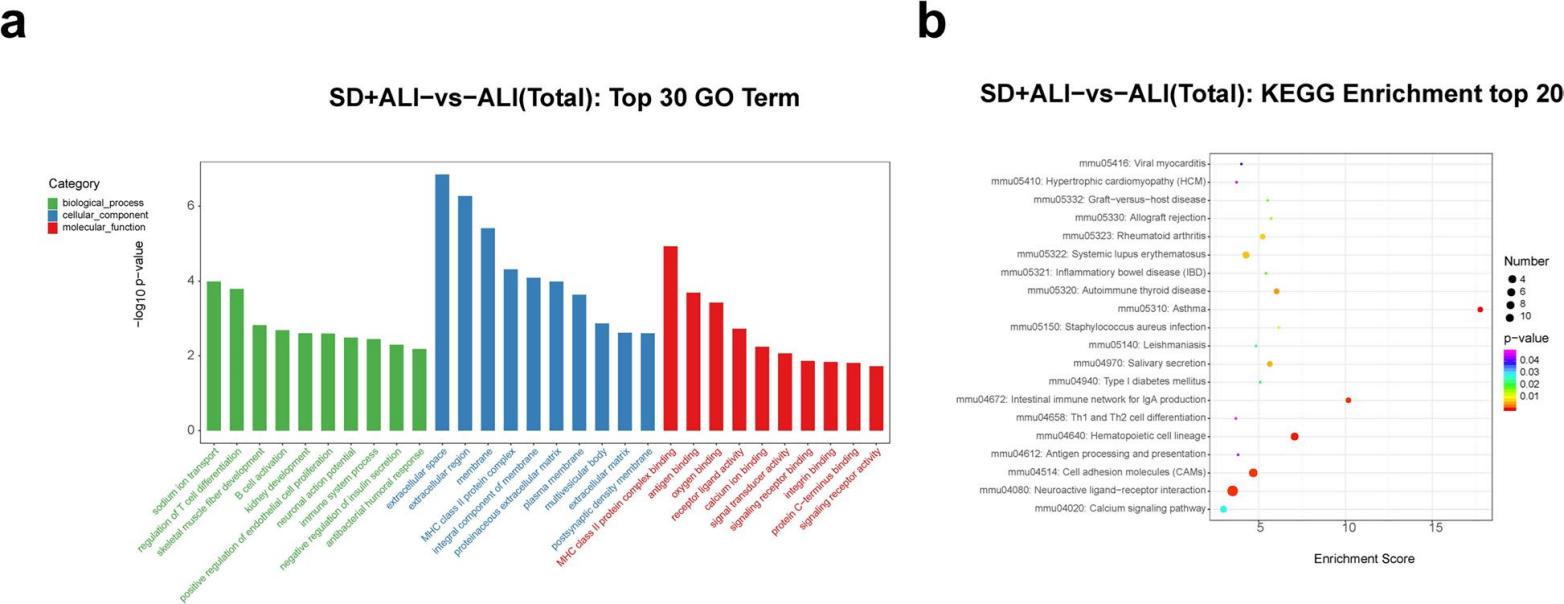
GO and KEGG analyses for the total dysregulated mRNAs in the SD + ALI group compared with the ALI group. **a** Top 30 enriched GO terms of the total dysregulated mRNAs were presented according to biological process (BP), cellular component (CC), and molecular function (MF). **b** Top 20 enriched KEGG of the total dysregulated mRNAs

Finally, GO functional analysis of the DE mRNAs in the SD + ALI group compared with the ALI group indicated that the most significantly enriched BP terms were sodium-ion transport, regulation of T-cell differentiation, B-cell activation, and positive regulation of endothelial cell proliferation. The main CC terms included extracellular space, extracellular region, membrane, and major histocopatibility complex (MHC) class II protein complex. The main MF terms were MHC class II protein complex binding, antigen binding, oxygen binding, and receptor–ligand activity (Fig. 11a). KEGG analysis indicated that the asthma, hematopoietic cell lineage, neuroactive ligand–receptor interaction, intestinal immune network for IgA production, and cell adhesion molecule(CAM) pathways were significantly enriched (Fig. 11b).

### Construction of a circRNA/lncRNA–miRNA–mRNA regulatory network based on DE circRNAs/lncRNAs, DE miRNAs, and DE mRNAs

**Fig. 12:**
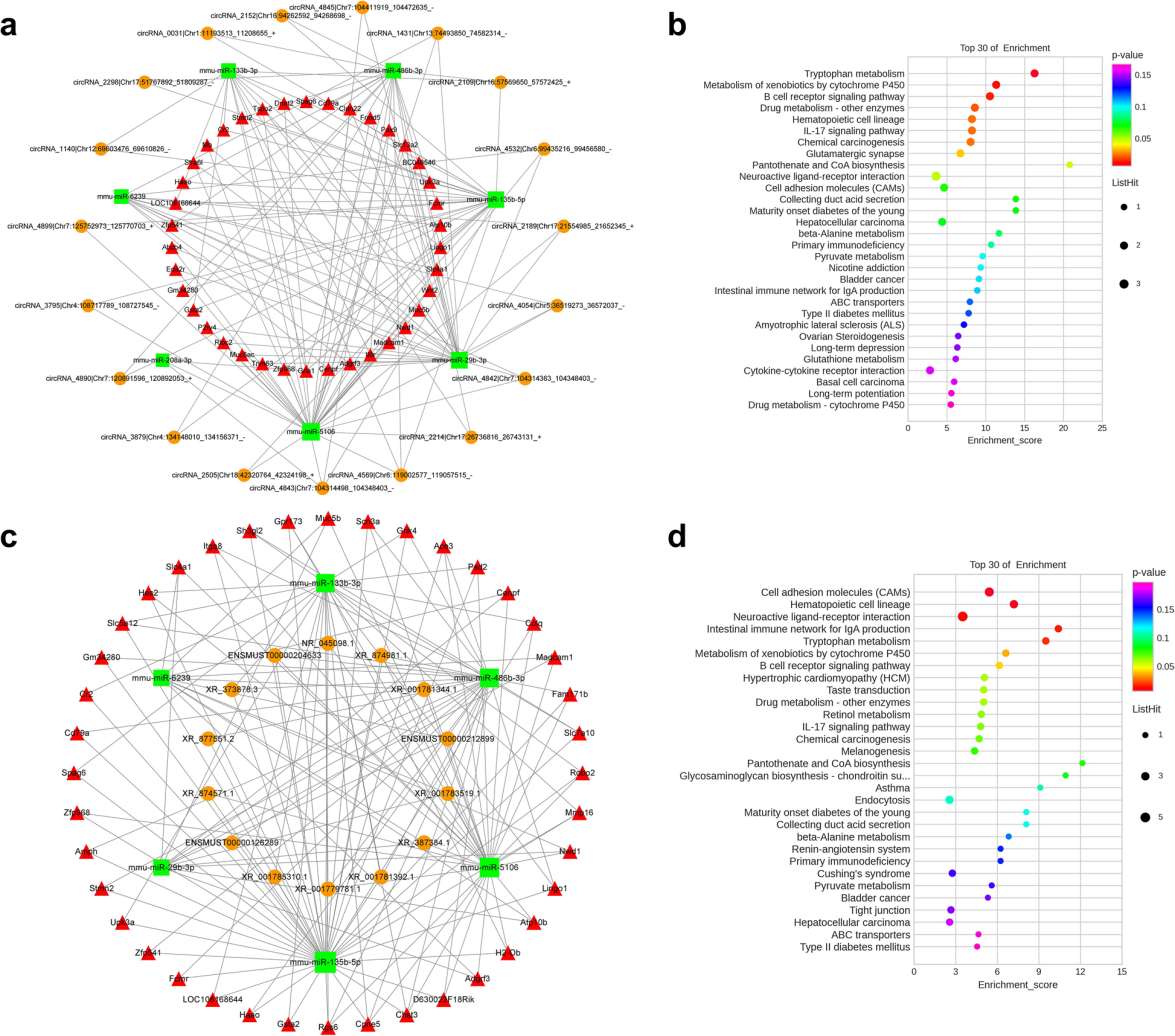
Established circRNA–miRNA–mRNA or lncRNA–miRNA–mRNA network in SD + ALI group compared with the ALI group. **a** Illustration of the circRNA–miRNA–mRNA network. The red triangle nodes represented DE mRNAs, the green rectangle nodes represented DE miRNAs, and the orange circle nodes represented DE circRNAs. **b** Top 30 enriched KEGG pathway enrichment of the DE mRNAs associated with the circRNA–miRNA–mRNA network. **c** Illustration of the lncRNA–miRNA–mRNA network. The red triangle nodes represented DE mRNAs, the green rectangle nodes represented DE miRNAs, and the orange circle nodes represented DE lncRNAs. **d** Top 30 enriched KEGG pathway enrichment of the DE mRNAs associated with the lncRNA–miRNA–mRNA network

According to the possible binding sites of miRNAs and mRNAs, the miRanda algorithm was used to predict the relationships between miRNAs and mRNAs. Compared with the ALI group, the SD+ ALI group exhibited 292 miRNA–mRNA interaction pairs and 280 miRNA–circRNA interaction pairs. ceRNA scores were calculated to construct a circRNA–miRNA–mRNA network, which consisted of 7 miRNAs, 19 circRNAs, and 38 mRNAs (Fig. 12a); The lncRNA–miRNA–mRNA network consisted of 6 miRNAs, 14 lncRNAs, and 40 mRNAs (Fig. 12c).

KEGG analysis of the SD + ALI group circRNA–miRNA–mRNA network revealed enrichment of the B-cell receptor signaling pathway, IL-17 signaling pathway, neuroactive ligand–receptor interaction, CAM, primary immunodeficiency, and cytokine–cytokine receptor interaction terms, among others (Fig. 12b). KEGG analyses of the lncRNA-miRNA-mRNA network also revealed inflammation–related signaling pathways (Fig. 12d).

###  RT-qPCR verification of DE miRNAs and DE circRNAs

**Fig. 13:**
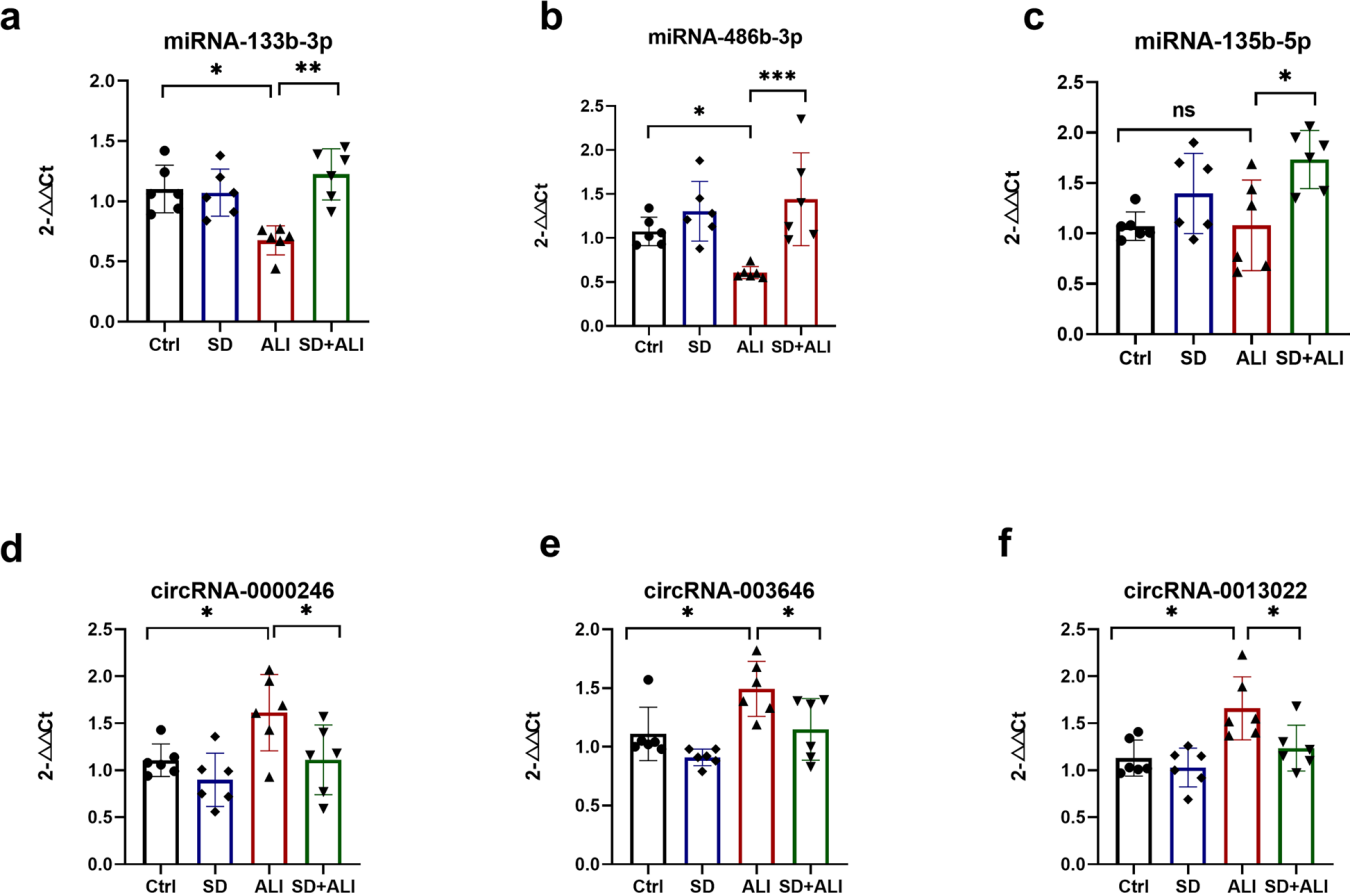
Several DE miRNA and circRNAs were obtained from the whole transcriptome and validated by RT-qPCR. The miRNA-133b-3p (**a**) or miRNA-486b-3p (**b**) relative expression level decreased in the ALI group compared with the Ctrl group, and increased in the SD + ALI group compared with the ALI group. **c** The miRNA-135b-5p relative expression level showed no difference between the Ctrl group and the ALI group, but increased in the SD + ALI group compared with the ALI group. The circRNA-0000246 (**d**), circRNA-003646 (**e**), or circRNA-0013022 relative expression level increased in the ALI group compared with the Ctrl group, and decreased in the SD + ALI group compared with the ALI group. Data represent the mean ± sd. *p < 0.05; **p < 0.01; ***p < 0.001; NS, no statistically significant difference

The results of RT-qPCR confirmed the DE miRNAs and DE circRNAs identified by sequencing. The expression levels of miRNA-133b-3p (Fig. 13a) and miR-486b-3p (Fig. 13b) were significantly lower in the ALI group than in the Ctrl group. In comparison, the expression levels of miRNA-133b-3p (Fig. 13a), miR-486b-3p (Fig. 13b) and miRNA-135b-5p (Fig. 13c) were significantly higher in the SD + ALI group than in the ALI group. The expression levels of circRNA-0000246 (Fig. 13d), circRNA-003646 (Fig. 13e), and circRNA-0013022(Fig. 13f) were consistent with the sequencing results. KEGG enrichment of the 3 circRNAs indicated that they may be associated with inflammatory signaling pathways (data not show).

## Discussion

ALI/ARDS still lacks effective treatment methods, and the mortality rate is as high as 40% [18]. Sepsis is a common cause of ALI/ARDS and is a significant public health problem worldwide [19]. Exploring the pathogenesis of sepsis-induced ALI is of great significance for finding more effective therapeutic targets. Many studies have shown that the central nervous system can regulate peripheral immunity [20, 21], and the peripheral nervous system also plays a vital role in regulating the inflammatory process in vivo [22, 23]. However, the roles of sympathetic nerves in regulating inflammation, especially lung inflammation, are still unclear. It is necessary to explore the deeper regulatory mechanism of sympathetic nerves in ALI. By establishing a mouse model of lung sympathetic denervation and a sepsis-induced ALI model, and performing high-throughput sequencing, we revealed a new mechanism of sympathetic nerves in ALI/ARDS at the transcriptome level.

6-OHDA has sympathetic toxicity, and is widely used to generate models of sympathetic denervation. In a previous study by our group, 6-OHDA was used to remove sympathetic nerves from adipose tissue [15], and other researchers have also confirmed that intranasally injection of 6-OHDA can remove most of the sympathetic nerves in the lungs [24]. In our study, Western blotting showed that the expression of TH decreased after intratracheal 6-OHDA administration. TH is the rate-limiting enzyme for catecholamine synthesis in sympathetic nerves. Moreover, the changing trend of NE in the lungs was consistent with that of TH. The above results showed that we successfully established a mouse lung SD model using 6-OHDA.

LPS, which is derived from gram-negative bacilli cell walls, can activate the body’s immune response and induce sepsis and even septic shock [25]. I.p. injection of LPS is often used to generate sepsis-induced ALI [26, 27]. In our study, at 8 hours i.p. injection of LPS, lung tissues from mice in the ALI group were subjected to HE staining. The lung injury score was higher in the ALI group than in the Ctrl group. In addition, the levels of TNF-α in serum and BALF were higher in the ALI group than in the Ctrl group, and the NF-κB signaling pathway in the lung tissues of mice in the ALI group was significantly enhanced. These data are consistent with ALI models in other studies [28], suggesting that i.p. injection of LPS successfully created a sepsis-induced ALI model. After SD, the lung injury score decreased, the NF-κB signaling pathway was inhibited, and the level of TNF-α in BALF decreased, suggesting that the removal of lung sympathetic nerves may attenuate sepsis-induced ALI by inhibiting NF-κB signaling. Sympathetic nerves act on corresponding adrenergic receptors by releasing catecholamines such as epinephrine and norepinephrine and participate in regulating sympathetic nerve inflammation in peripheral organs. A variety of cells in and outside the lung, including smooth muscle cells, glandular cells, immune cells, etc., can express adrenergic receptors in a tissue-specific manner [29–31]. Previous studies have suggested that sympathetic nerves can promote pulmonary inflammation [32, 33], and that phagocyte-derived catecholamines can enhance inflammation [34, 35]. The mechanism by which sympathetic nerves participate in sepsis-induced ALI is unclear. Nevertheless, sympathetic nerve excitation plays a role in amplifying inflammatory responses in sepsis [36]. SD may alleviate sepsis-induced ALI, according to the current study.

NcRNAs play essential roles in ALI. To further explore the mechanism of sympathetic nerve regulation in ALI, we investigated whether sympathetic nerves affect circRNA, lncRNA, or miRNA levels during sepsis-induced ALI. We found many differentially expressed circRNAs, lncRNAs, miRNAs, and mRNAs in the ALI group, compared with the Ctrl group that regulated inflammatory signaling pathways indicated by GO and KEGG enrichment. Some previous studies have also used whole-transcriptome sequencing to assess the transcriptome changes in ALI and have proven that multiple circRNAs, lncRNAs, miRNAs, and mRNAs are significantly altered in ALI [16, 17]. In the current study, we identified 629 DE circRNAs, 269 DE lncRNAs, 192 DE mRNAs, and 7 DE circRNAs in the SD + ALI group compared with the ALI group. We performed GO and KEGG enrichment analyses to further understand the roles of these DE ncRNA and DE mRNAs. GO and KEGG enrichment analyses of the DE ncRNAs and DE mRNAs in the ALI group showed that many immune-inflammatory processes and pathways included the NF-κB signaling pathway (Fig. S2, S3, S4, S5). Surprisingly, many DE circRNAs, DE lncRNAs, DE miRNAs, and DE mRNAs (Fig. 4d, S1c, S1f) were found in the SD + ALI group compared with the ALI group, too. GO and KEGG analyses identified many inflammatory and immune responses such as the TGF-β signaling pathway, TNF signaling, MAPK signaling, Th17 cell differentiation, and B-cell receptor signaling pathways, between the DE ncRNAs and the DE mRNAs. Interestingly, some miRNAs were downregulated in ALI mice but, were upregulated in SD + ALI mice, such as miRNA-133b-3p and miR-486b-3p. Many circRNAs or lncRNAs (data not shown) exhibited patterns similar to those of miRNAs, such as the circRNAs mmu_circ_0000246, mmu_circ_003646, and mmu_circ_0013022. Some of these miRNAs and circRNAs were verified by RT–qPCR, suggesting that sympathetic nerves may affect the expression of these ncRNAs in sepsis-induced ALI. Finally, based on these DE circRNAs/DE lncRNAs, DE miRNAs, and DE mRNAs, circRNA/lncRNA–miRNA–mRNA networks were constructed. KEGG analysis of the circRNA–miRNA–mRNA network showed that in SD + ALI group mice, a large number of inflammation-related signaling pathways were enriched, such as the B-cell receptor signaling pathway and IL-17 signaling pathway; the same phenomenon was observed in lncRNA–miRNA–mRNA network KEGG analysis. These findings suggest that sympathetic nerves may further affect sepsis-induced ALI by regulating the expression of ncRNAs, which reveals a new mechanism of sympathetic nerve regulation of ALI.

Compared with the ALI group, the most significant miRNA in the SD + ALI group was miR-135b-5p, suggesting that miRNA-135b-5p may change with sympathetic signaling and that elevated miR-135b-5p reduces poststroke nervous system inflammation [37]. Many miRNAs found in our research were reported to be involved in the regulation of ALI. For instance, miR-135b-5p [38], miR-486-5p [39], miR-155 [40], miR-139-5p [41], and miR-499-5p [42] have been shown to regulate ALI by targeting different proteins and signaling pathways, and miRNA-486-5p may be a potential diagnostic biomarker for sepsis [43]. Some miRNAs have been reported to regulate inflammation; for example, miRNA-208-5p regulates myocardial injury in septic mice [44], and miRNA-29 promotes pancreatic β cell inflammation via TRAF3 [45]. CircRNAs or lncRNAs, which act as miRNAs, were significantly differentially expressed in the ALI group in this study and the previous study of ALI [46, 47]. It has also been recognized that circANKR inhibits the NF-κB signaling pathway by acting on the miR-31/MyD88 axis [48], and lncRNA TUG1 also alleviates sepsis-induced acute lung injury by targeting miRNA-34b-5p [49]. These results indicated that some circRNAs or lncRNAs play important roles in ALI, similar to miRNAs; Although the GO and KEGG enrichment analyses suggest that most DE circRNAs and DE lncRNAs found in this study are associated with inflammation, the roles of them in ALI are unclear, and they need to be further studied in the future.

Sympathetic nerves participate in several pathophysiological processes by affecting the expression of ncRNAs. NE downregulated miR-19a-3p in vitro [10], while SD can improve cardiac ischemia–reperfusion in rats and affect the expression of mRNAs, miRNAs, lncRNAs, and circRNAs in the thoracic sympathetic trunk [50]. Sympathetic nerves can transmit miRNA-181a, which is involved in the regulation of hypertension [11], and postmyocardial infarction cardiac insufficiency can be improved by miRNA [51]. MiR-21 is involved in sympathetically mediated apoptosis of ovarian granulosa cells [52]. In turn, miRNAs can affect the signaling levels of sympathetic nerves [10, 53, 54]. In addition to participating in the regulation of sympathetic inflammation, miRNAs participate in the cholinergic anti-inflammatory pathway [40, 55]. CircRNAs are also regulated by sympathetic nerves; Cai W et al. found that renal SD in patients with persistent hypertension affects the expression of circRNAs [56]. Interestingly, it seems that ncRNAs also participate in the activity of nerves. CircRNAs play roles in the development and pathological processes of the nervous system [57]; some circRNAs can even be directly translated into proteins during myogenesis [58]. Silencing lncRNAs may reduce NE levels in myocardial ischemia [59]. In conclusion, there is crosstalk between sympathetic nerves and ncRNAs during physiological and pathological processes. After SD, whole–transcriptome sequencing detected a large number of ncRNAs that may be involved in inflammation in the lungs in sepsis-induced ALI. The functional analysis of the DE ncRNAs suggested that the DE circRNAs, DE lncRNAs, or DE miRNAs may be the intermediate links in regulating sympathetic nerve involvement in lung inflammation.

## Conclusion

Our study found that SD can alleviate sepsis-induced ALI by inhibiting the NF-κB signaling pathway and reducing the level of TNF-α in BALF. Through whole-transcriptome sequencing, we found that SD can affect the expression of ncRNAs, such as circRNAs, lncRNAs, and miRNAs, in the lungs of mice. The expression of circRNAs, lncRNAs, miRNAs, and mRNAs in the lungs of SD + ALI mice was further analyzed by KEGG and GO analysis. Many inflammatory signaling pathways were affected, and the significance of the differential expression was further illustrated by further construction of circRNA/lncRNA–miRNA–mRNA networks. The findings indicate that sympathetic nerves may regulate the inflammatory response to lung injury by affecting the mechanisms of ncRNAs, providing clues for further exploring the roles of sympathetic nerves in regulating inflammation in the lungs and shedding light on ALI therapy. Admittedly, the main limitation of the present study is that we only used male mice for the preliminary study, and the sample size was too small. Moreover, there is not enough analysis to narrow the list of DE RNAs as key regulators, and there is no deeper analysis to look into DE RNAs. In order to find therapeutic targets related to sympathetic nerves, how sympathetic nerves affect the expression of ncRNAs through neural signaling needs to be further clarified. In addition, the roles of specific DE circRNAs, lncRNAs or miRNAs in sympathetic nerve regulation of the lungs and the particular mechanism of action of the injury need to be elucidated.

## Supplementary Information

**Additional file 1: Fig. S1.** The survival proportions in the Ctrl, SD, ALI, and SD + ALI group. **Fig. S2.** The distinct mRNAs expression profiles between groups. **a** The heat map represents hierarchical clustering for DE mRNAs between the SD group and Ctrl group; **b** The heat map represents hierarchical clustering for DE mRNAs between the ALI group compared with the Ctrl group; **c** The heat map represents hierarchical clustering for DE mRNAs between the SD + ALI group compared with the ALI group. **d** The volcano plots for DE mRNAs between SD group and Ctrl group; **e** The volcano plots for DE mRNAs between the ALI group compared with the Ctrl group; **f** The volcano plots for DE mRNAs between the SD + ALI group compared with the ALI group. Up-regulated expression was indicated as “red”, and down-regulated expression was indicated as “green”. **Fig. S3.** GO and KEGG analyses for the total dysregulated circRNAs in the ALI group compared with the Ctrl group. **a** Top 30 enriched GO terms of the total dysregulated circRNAs were presented according to biological process (BP), cellular component (CC), and molecular function (MF). **b** Top 20 enriched KEGG pathways of the total dysregulated circRNAs. **Fig. S4.** GO and KEGG analyses for the total dysregulated lncRNAs in the ALI group compared with the Ctrl group. **a** Top 30 enriched GO terms of the total dysregulated lncRNAs were presented according to biological process (BP), cellular component (CC), and molecular function (MF). **b** Top 20 enriched KEGG pathways of the total dysregulated lncRNAs. **Fig. S5.** GO and KEGG analyses for the total dysregulated miRNAs in the ALI group compared with the Ctrl group. **a** Top 30 enriched GO terms of the total dysregulated miRNAs were presented according to biological process (BP), cellular component (CC), and molecular function (MF). **b** Top 20 enriched KEGG pathways of the total dysregulated miRNAs. **Fig. S6.** GO and KEGG analyses for the total dysregulated mRNAs in the ALI group compared with the Ctrl group. **a** Top 30 enriched GO terms of the total dysregulated mRNAs were presented according to biological process (BP), cellular component (CC), and molecular function (MF). **b** Top 20 enriched KEGG pathways of the total dysregulated mRNAs.

**Additional file 2.**

## Data Availability

The datasets presented in our study can be found in online repositories. Data are available at NCBI Sequence Read Archive (SRA): BioProject: PRJNA850459.

## Abbreviations

6-OHDA: 6-hydroxydopamine
TH: Tyrosine hydroxylase
SD: Sympathetic denervation
ALI: Acute lung injury
NE: Norepinephrine
TNF-α: Tumor necrosis factor-α
BALF: Bronchoalveolar lavage fluid
DE: Differently expression
ncRNAs: non-coding RNAs
circRNAs: circle RNAs
lncRNAs: long noncoding RNAs
miRNAs: microRNAs
GO: Gene Ontology
KEGG: Kyoto Encyclopedia of Genes and Genomes
ECM: Extracellular matrix
ERK: Extracellular regulated protien kinases
TGF-β: Transforming growth factor-β
MHC: Major histocopatibility complex
CAM: Cell Adhesion molecule
