# Supplementary material for: Deregulated RNAs involved in sympathetic regulation of sepsis-induced acute lung injury based on whole transcriptome sequencing
Source: BMC Genomics. 2022 Dec 16;23:836. doi: 10.1186/s12864-022-09073-8 (PMC9758828; doi:10.1186/s12864-022-09073-8)
Supplement: Supplementary file 1 — Additional file 1: Fig. S1. The survival proportions in the Ctrl, SD, ALI, and SD + ALI group. Fig. S2. The distinct mRNAs expression profiles between groups. a The heat map represents hierarchical clustering for DE mRNAs between the SD group and Ctrl group; b The heat map represents hierarchical clustering for DE mRNAs between the ALI group compared with the Ctrl group; c The heat map represents hierarchical clustering for DE mRNAs between the SD + ALI group compared with the ALI group. d The volcano plots for DE mRNAs between SD group and Ctrl group; e The volcano plots for DE mRNAs between the ALI group compared with the Ctrl group; f The volcano plots for DE mRNAs between the SD + ALI group compared with the ALI group. Up-regulated expression was indicated as “red”, and down-regulated expression was indicated as “green”. Fig. S3. GO and KEGG analyses for the total dysregulated circRNAs in the ALI group compared with the Ctrl group. a Top 30 enriched GO terms of the total dysregulated circRNAs were presented according to biological process (BP), cellular component (CC), and molecular function (MF). b Top 20 enriched KEGG pathways of the total dysregulated circRNAs. Fig. S4. GO and KEGG analyses for the total dysregulated lncRNAs in the ALI group compared with the Ctrl group. a Top 30 enriched GO terms of the total dysregulated lncRNAs were presented according to biological process (BP), cellular component (CC), and molecular function (MF). b Top 20 enriched KEGG pathways of the total dysregulated lncRNAs. Fig. S5. GO and KEGG analyses for the total dysregulated miRNAs in the ALI group compared with the Ctrl group. a Top 30 enriched GO terms of the total dysregulated miRNAs were presented according to biological process (BP), cellular component (CC), and molecular function (MF). b Top 20 enriched KEGG pathways of the total dysregulated miRNAs. Fig. S6. GO and KEGG analyses for the total dysregulated mRNAs in the ALI group compared with the Ctrl group. a Top 30 enr [file 12864_2022_9073_MOESM1_ESM.docx]

**Additional file 1**


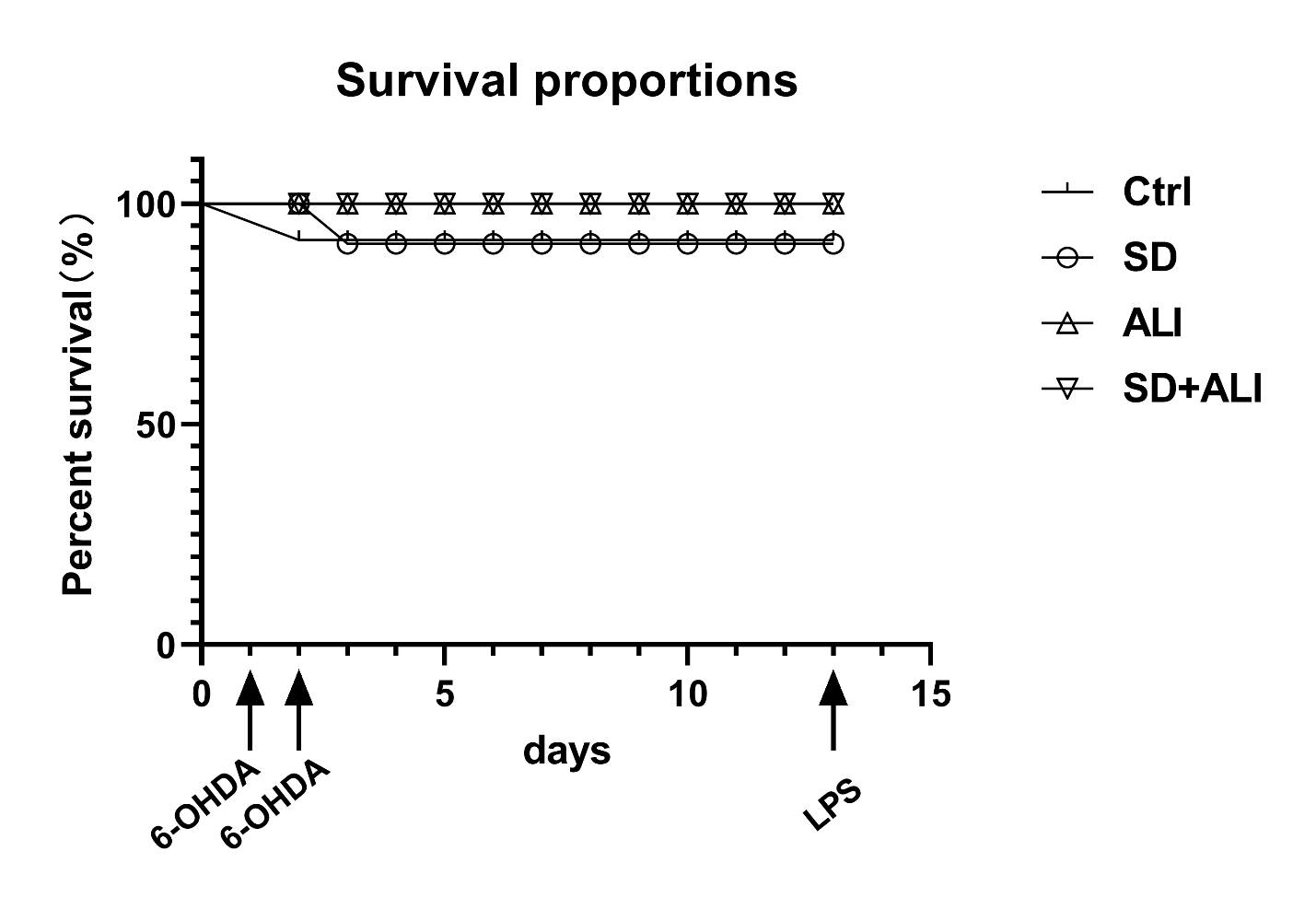


**Figure. S1** The survival proportions in the Ctrl, SD, ALI, and SD+ALI group.


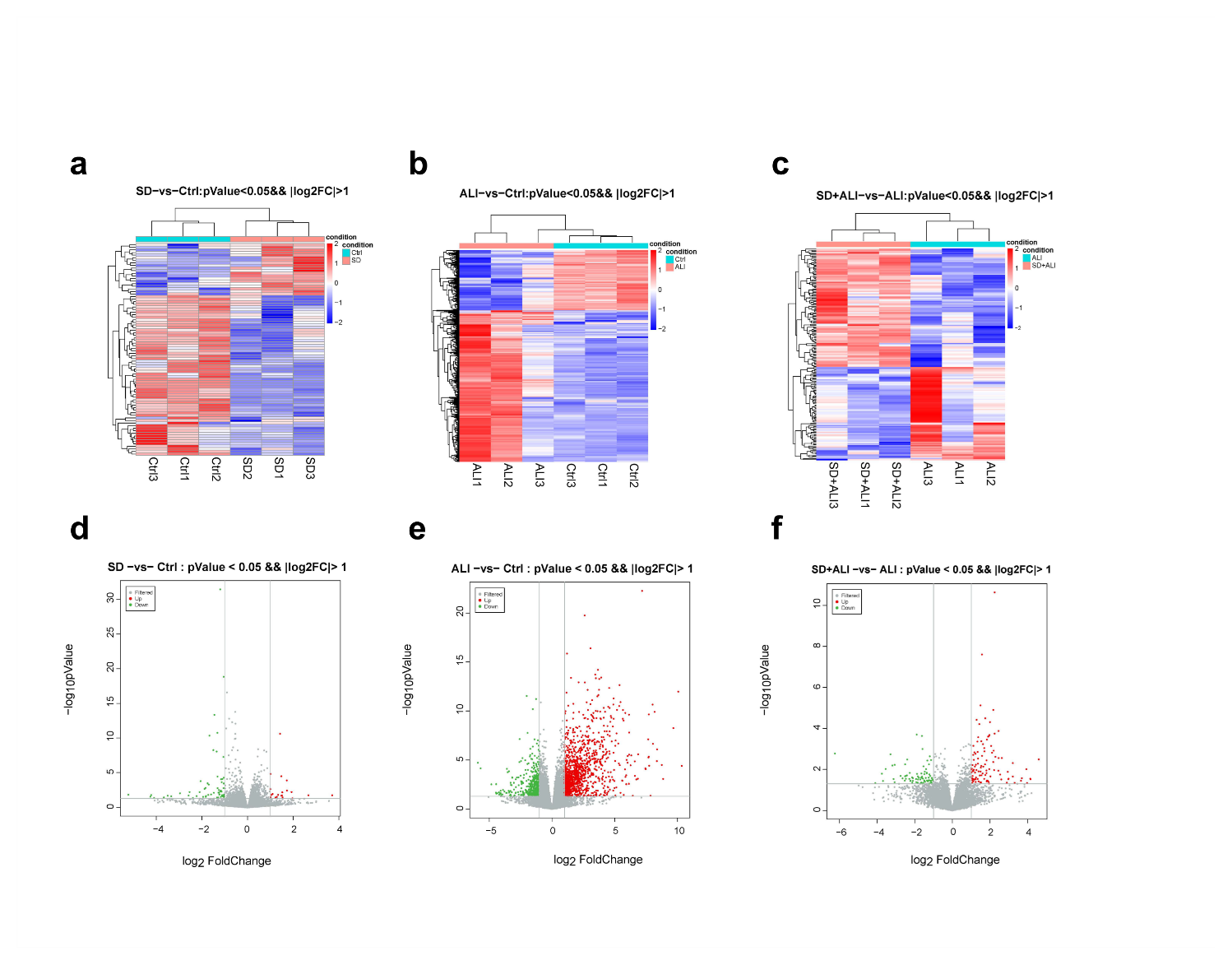


**Figure. S2** The distinct mRNAs expression profiles between groups. **a** The heat map represents hierarchical clustering for DE mRNAs between the SD group and Ctrl group; **b** The heat map represents hierarchical clustering for DE mRNAs between the ALI group compared with the Ctrl group; **c** The heat map represents hierarchical clustering for DE mRNAs between the SD+ALI group compared with the ALI group. **d** The volcano plots for DE mRNAs between SD group and Ctrl group; **e** The volcano plots for DE mRNAs between the ALI group compared with the Ctrl group; **f** The volcano plots for DE mRNAs between the SD+ALI group compared with the ALI group. Up-regulated expression was indicated as “red”, and down-regulated expression was indicated as “green”.

**
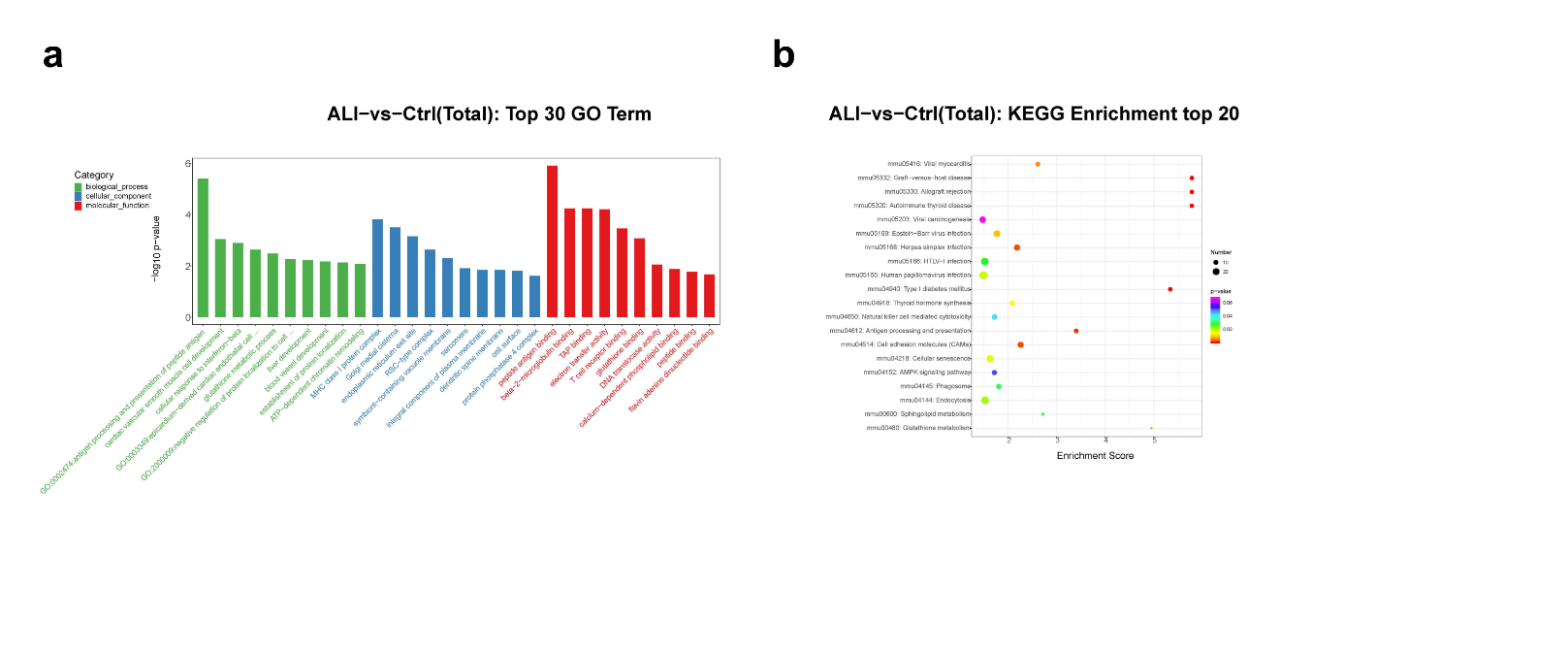
**

**Figure. S3** GO and KEGG analyses for the total dysregulated circRNAs in the ALI group compared with the Ctrl group. **a** Top 30 enriched GO terms of the total dysregulated circRNAs were presented according to biological process (BP), cellular component (CC), and molecular function (MF). **b** Top 20 enriched KEGG pathways of the total dysregulated circRNAs.

**
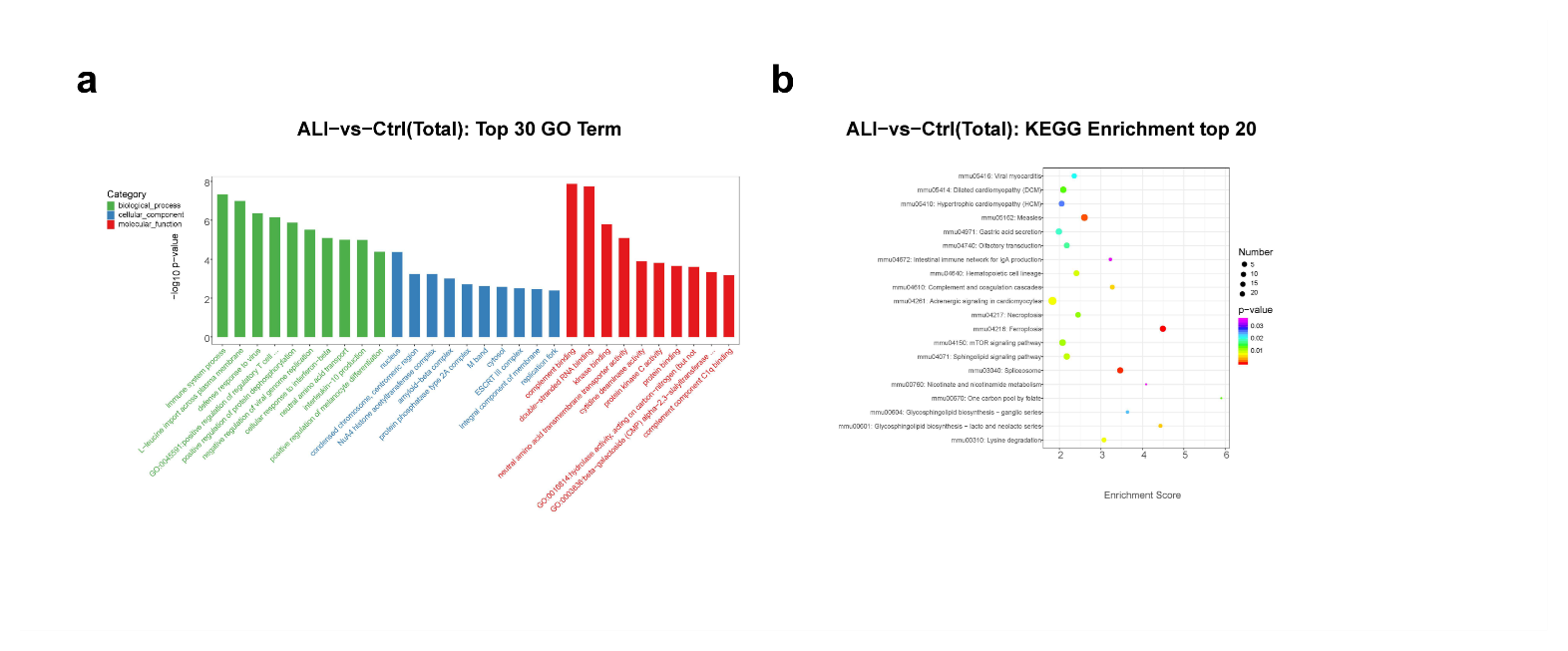
**

**Figure. S4** GO and KEGG analyses for the total dysregulated lncRNAs in the ALI group compared with the Ctrl group. **a** Top 30 enriched GO terms of the total dysregulated lncRNAs were presented according to biological process (BP), cellular component (CC), and molecular function (MF). **b** Top 20 enriched KEGG pathways of the total dysregulated lncRNAs.

**
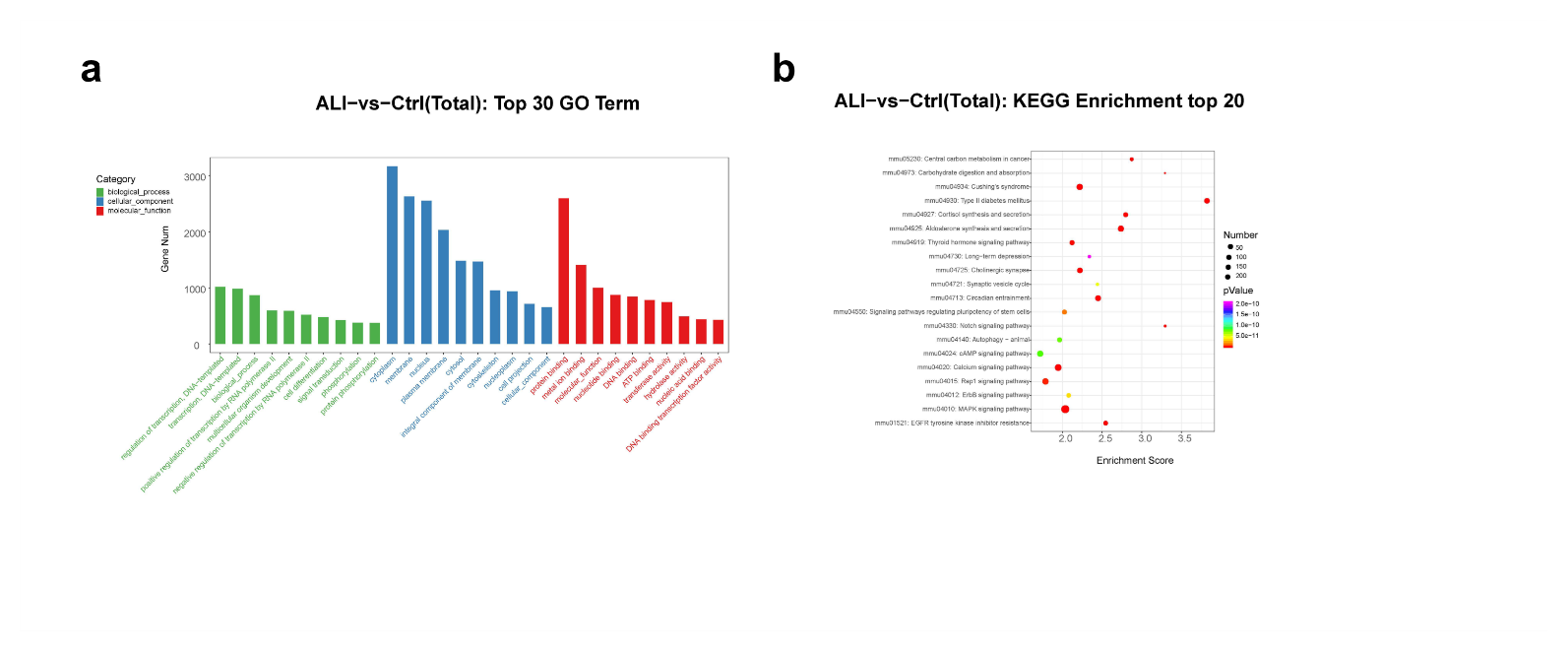
**

**Figure. S5** GO and KEGG analyses for the total dysregulated miRNAs in the ALI group compared with the Ctrl group. **a** Top 30 enriched GO terms of the total dysregulated miRNAs were presented according to biological process (BP), cellular component (CC), and molecular function (MF). **b** Top 20 enriched KEGG pathways of the total dysregulated miRNAs.

**
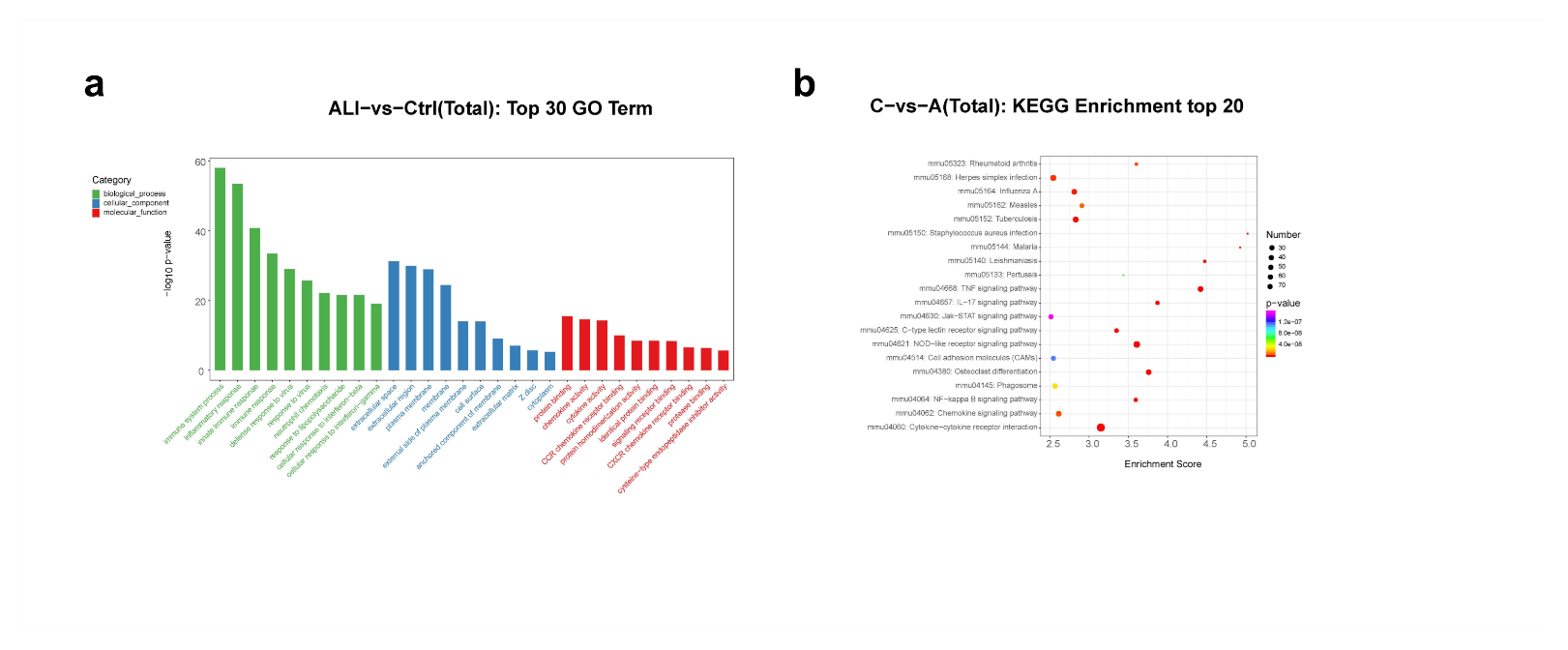
**

**Figure. S6** GO and KEGG analyses for the total dysregulated mRNAs in the ALI group compared with the Ctrl group. **a** Top 30 enriched GO terms of the total dysregulated mRNAs were presented according to biological process (BP), cellular component (CC), and molecular function (MF). **b** Top 20 enriched KEGG pathways of the total dysregulated mRNAs.

**Primers for RT-qPCR**

1. mmu-miR-133b-3p

F primer AGTTCTTGATTTGGTCCCCTTC

R primer AATGGTTGTTCTCCACTCTCTCTC

Size:70bp

1. mmu-miR-486b-3p

F primer ATTAGATTACGGGGCAGCTCAGT

R primer GTGCAGGGTCCGAGGT

Size:66bp

1. mmu-miR-135b-5p

F primer CCGCTCTATGGCTTTTCATTC

R primer TATGGTTTTGACGACTGTGTGAT

Size:66bp

1. mmu-circ-0000246

F primer TCCAAGGAACCAGTGTGAAGTAAT

R primer GAGCTGCATGGTCTGCTAACA

Size:66bp

1. mmu-circ-0003646

F primer GGAGCATCTCCTTTCACTGTCG

R primer TCCAGGGCAAGCACAATCT

Size:159bp

1. mmu-circ-0013022

F primer TGTACAGGAGGAAAACCAGCTG

R primer GCGATAGCATGCATCTGAAA

Size:104bp
