# Supplementary material for: Deregulated RNAs involved in sympathetic regulation of sepsis-induced acute lung injury based on whole transcriptome sequencing
Source: BMC Genomics. 2022 Dec 16;23:836. doi: 10.1186/s12864-022-09073-8 (PMC9758828; doi:10.1186/s12864-022-09073-8)

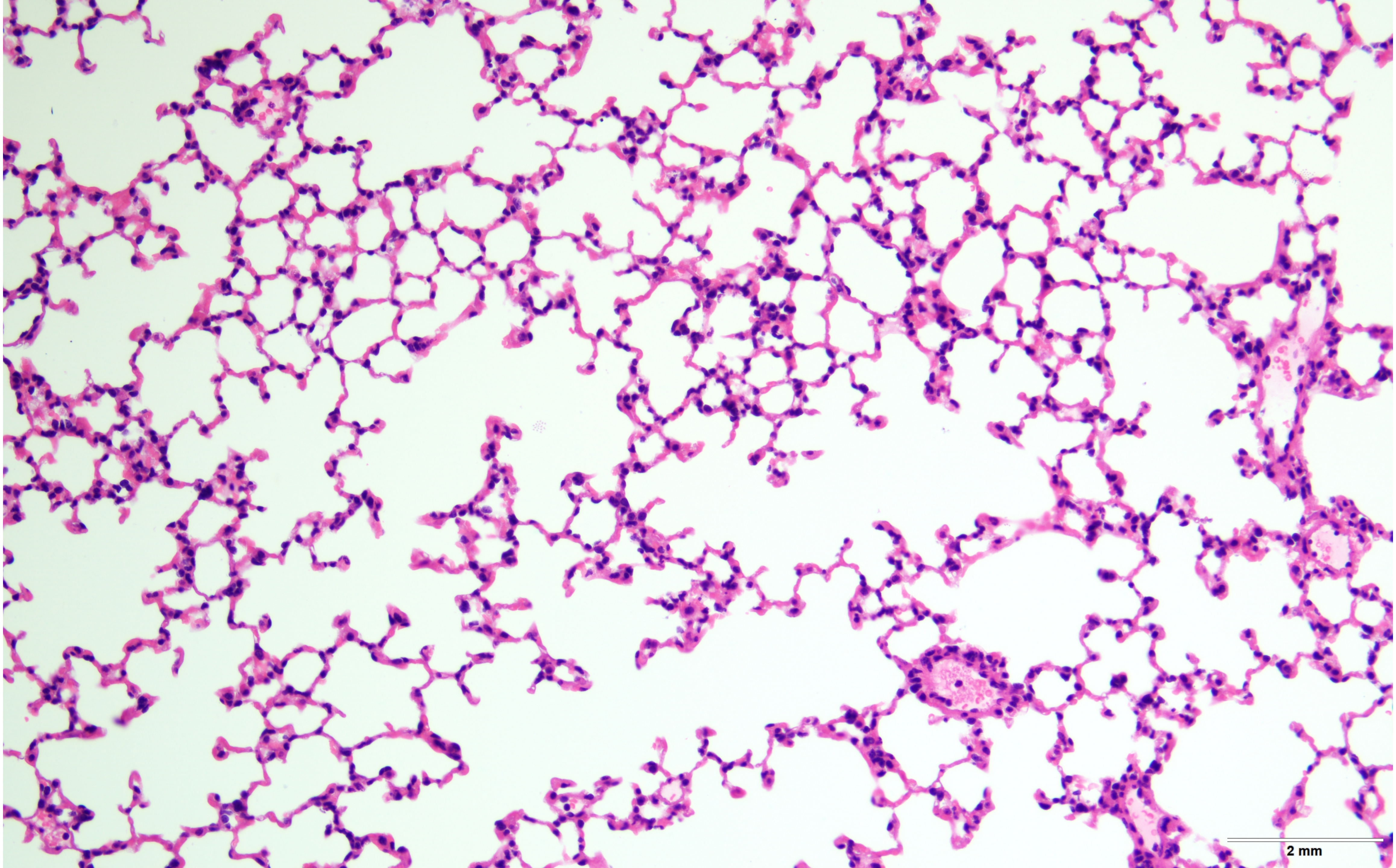
2 mm

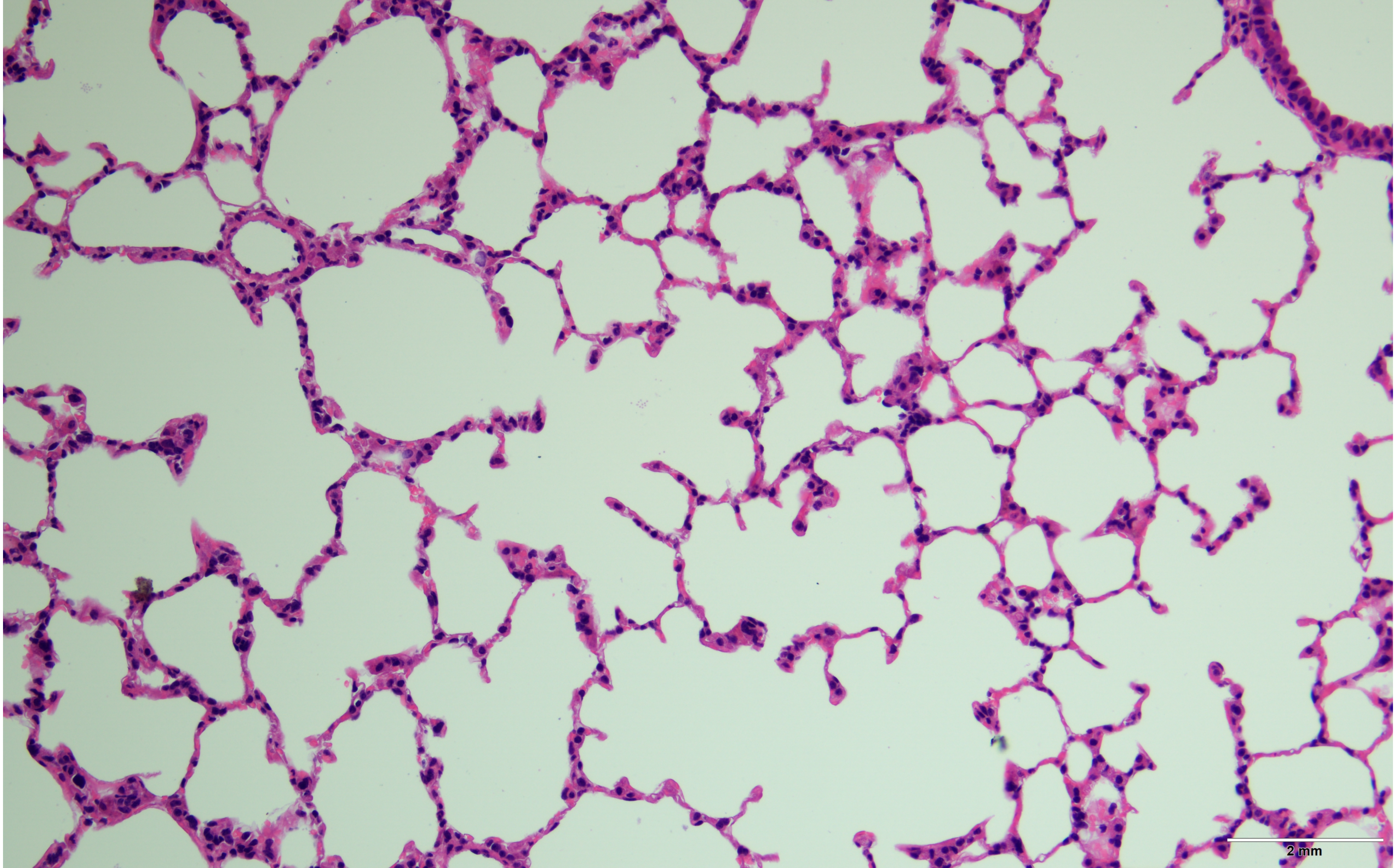
2 mm

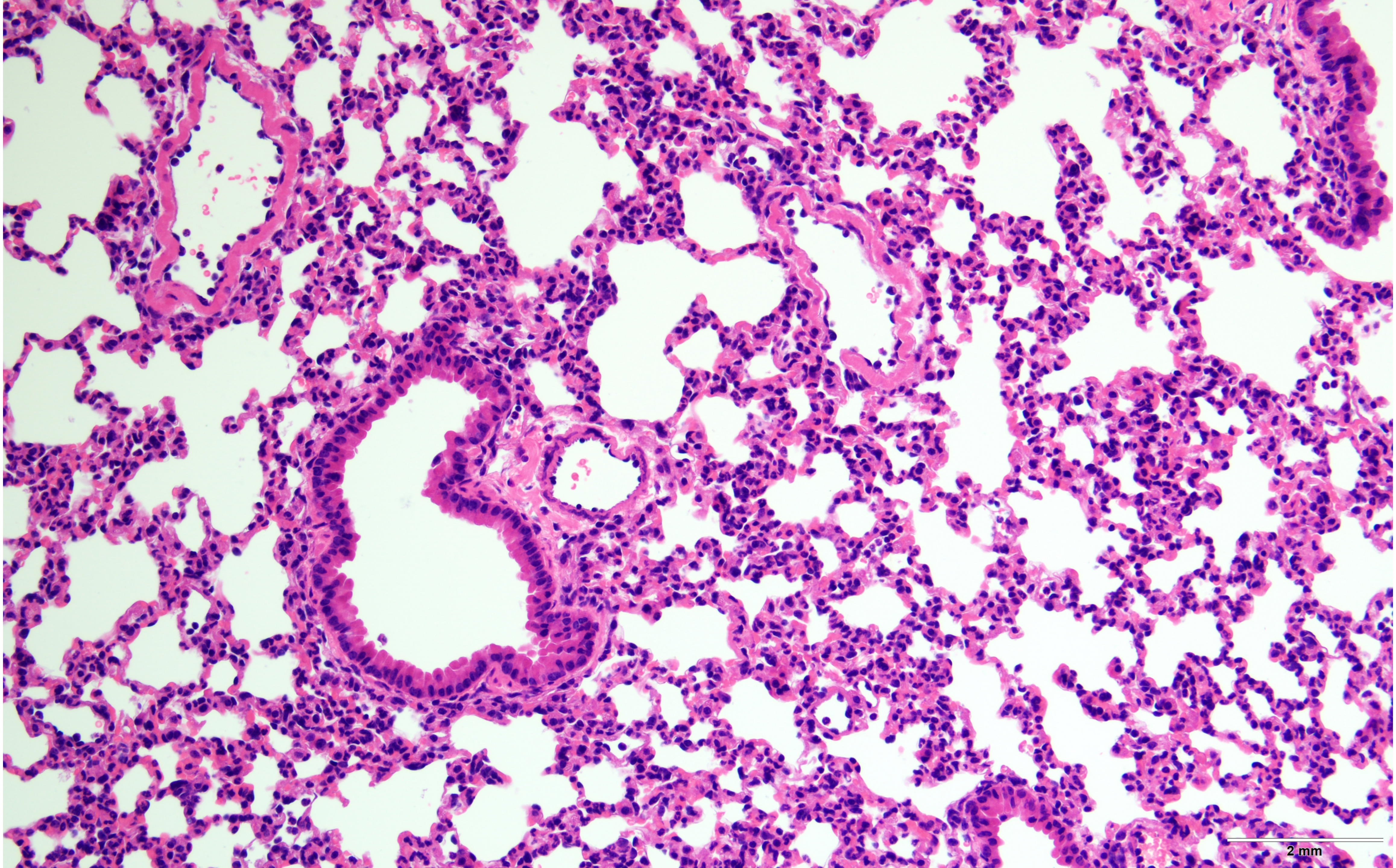
2 mm

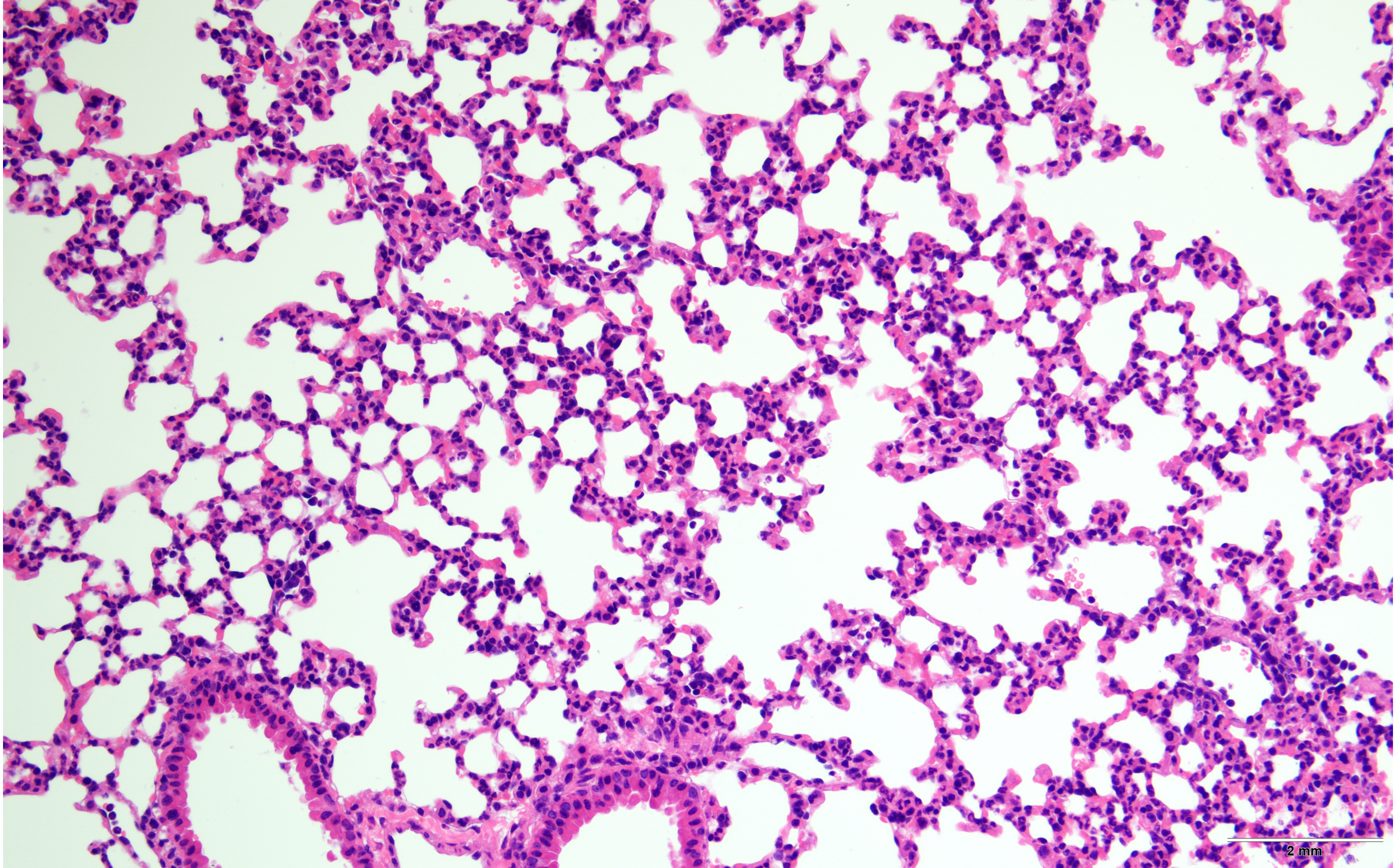
2 mm

WB Sample image used in in Figure 1

TH (60KDa)

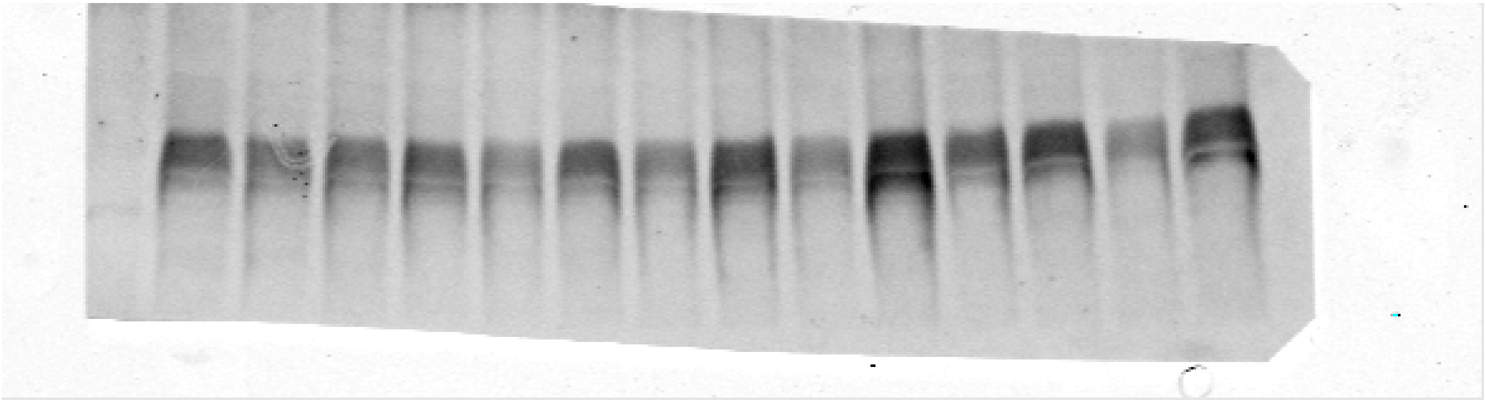

β-Actin (42KDa)

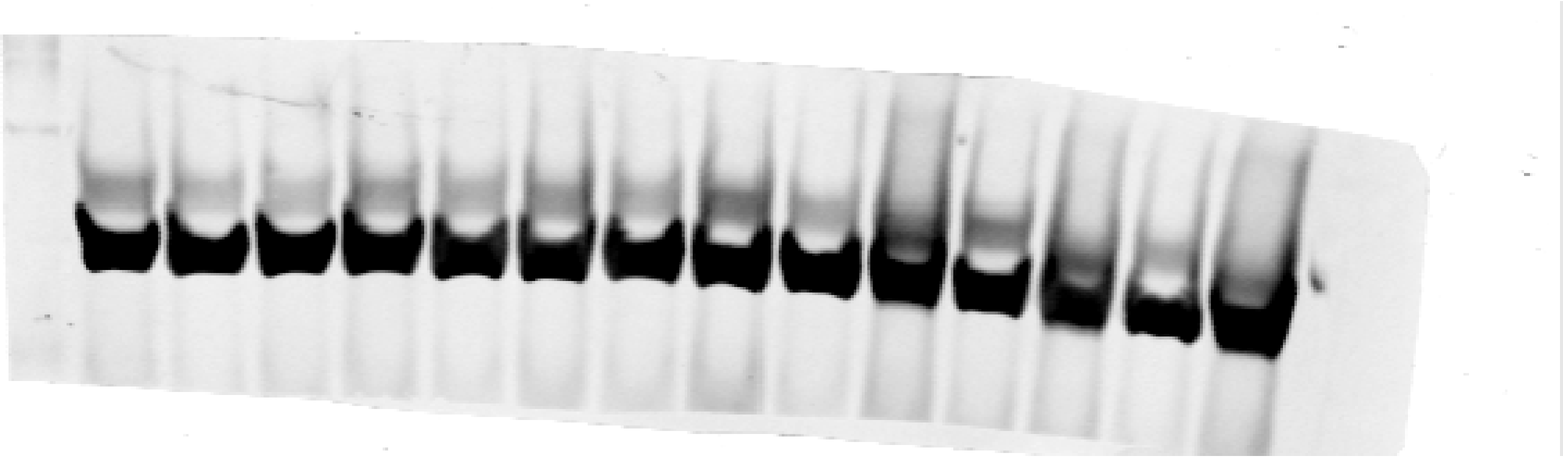

WB Sample image used in in Figure 3

p-IκBα (35 KDa)

IκBα (35 KDa)

β-Actin (42KDa)

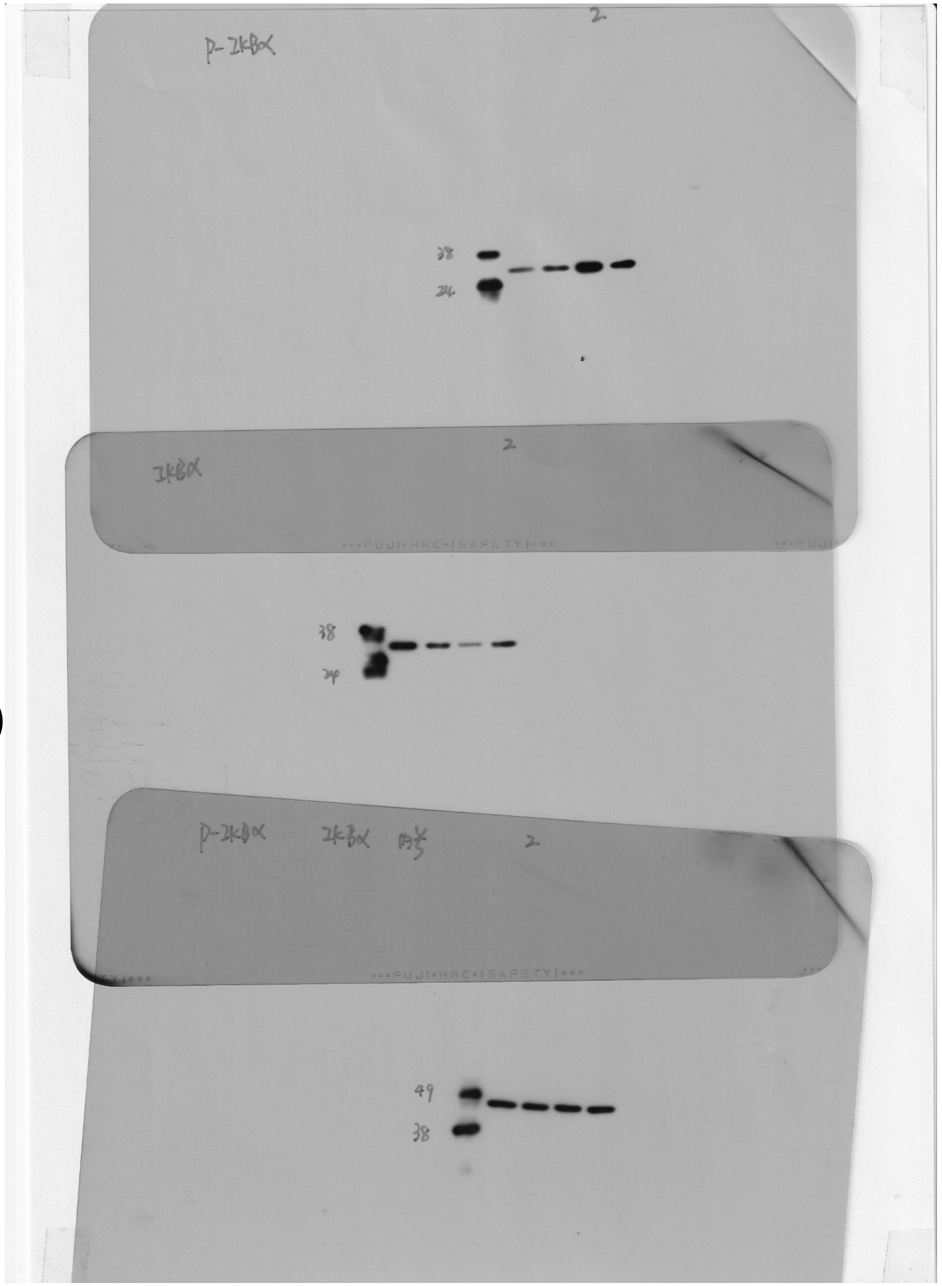

p-NFκB p65 (65 KDa)

NFκB P65 (65 KDa)

β-Actin (42KDa)

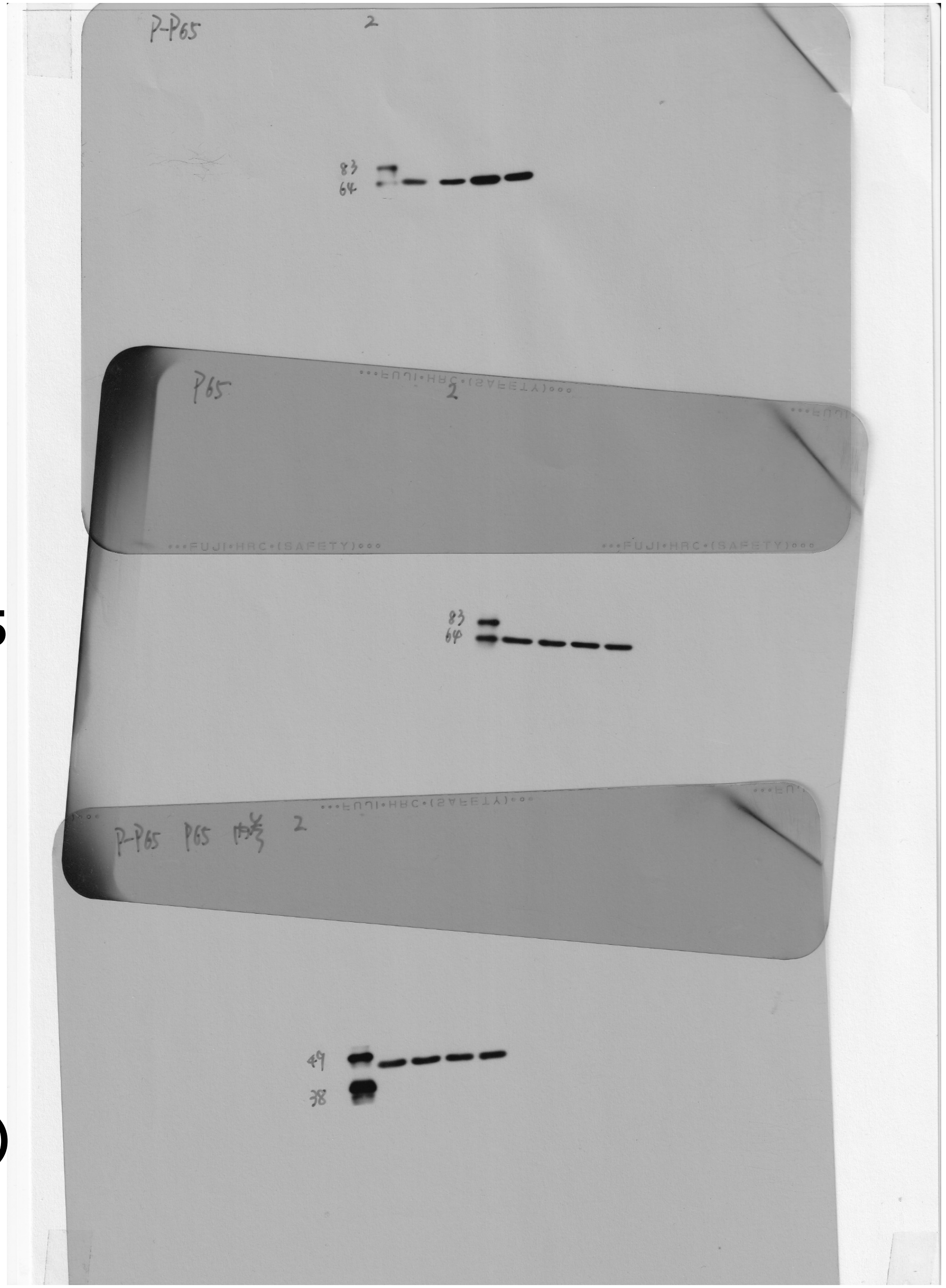

WB replicates image (p-NFκB p65, NFκB p65)

p-NFκB p65 (65 KDa)

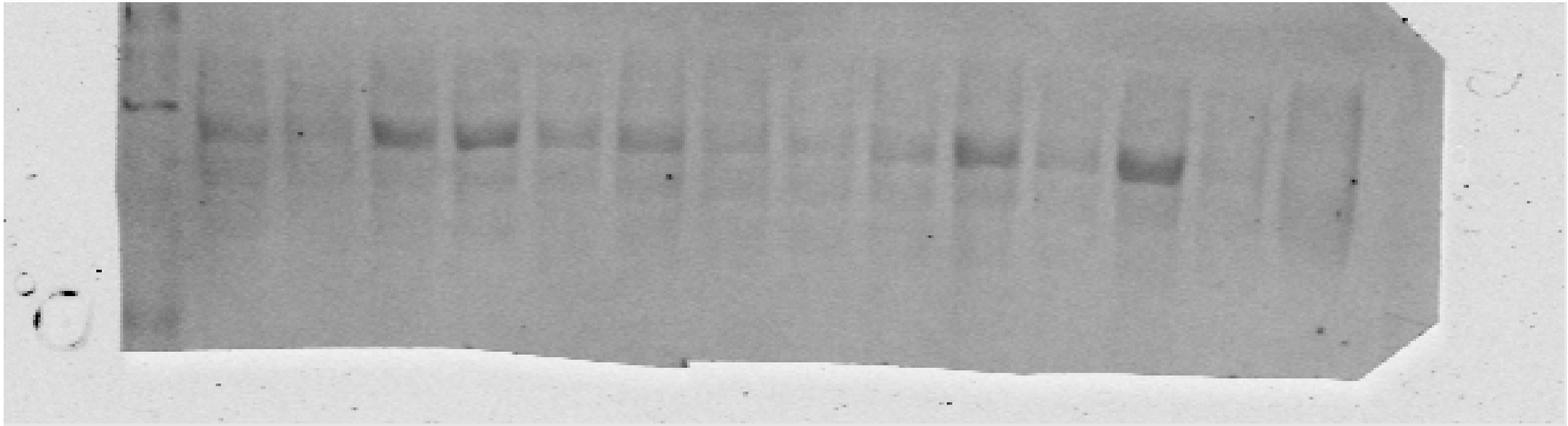

NFκB P65 (65 KDa)

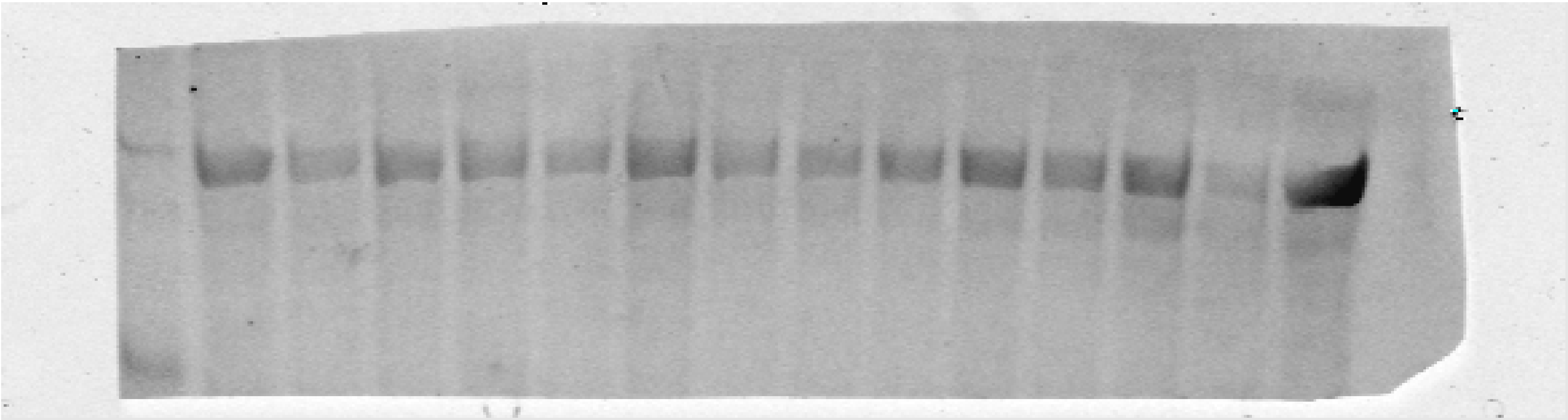

WB replicates image (p-IκBα ,IκBα p-NFκB p65, NFκB p65， β-Actin)

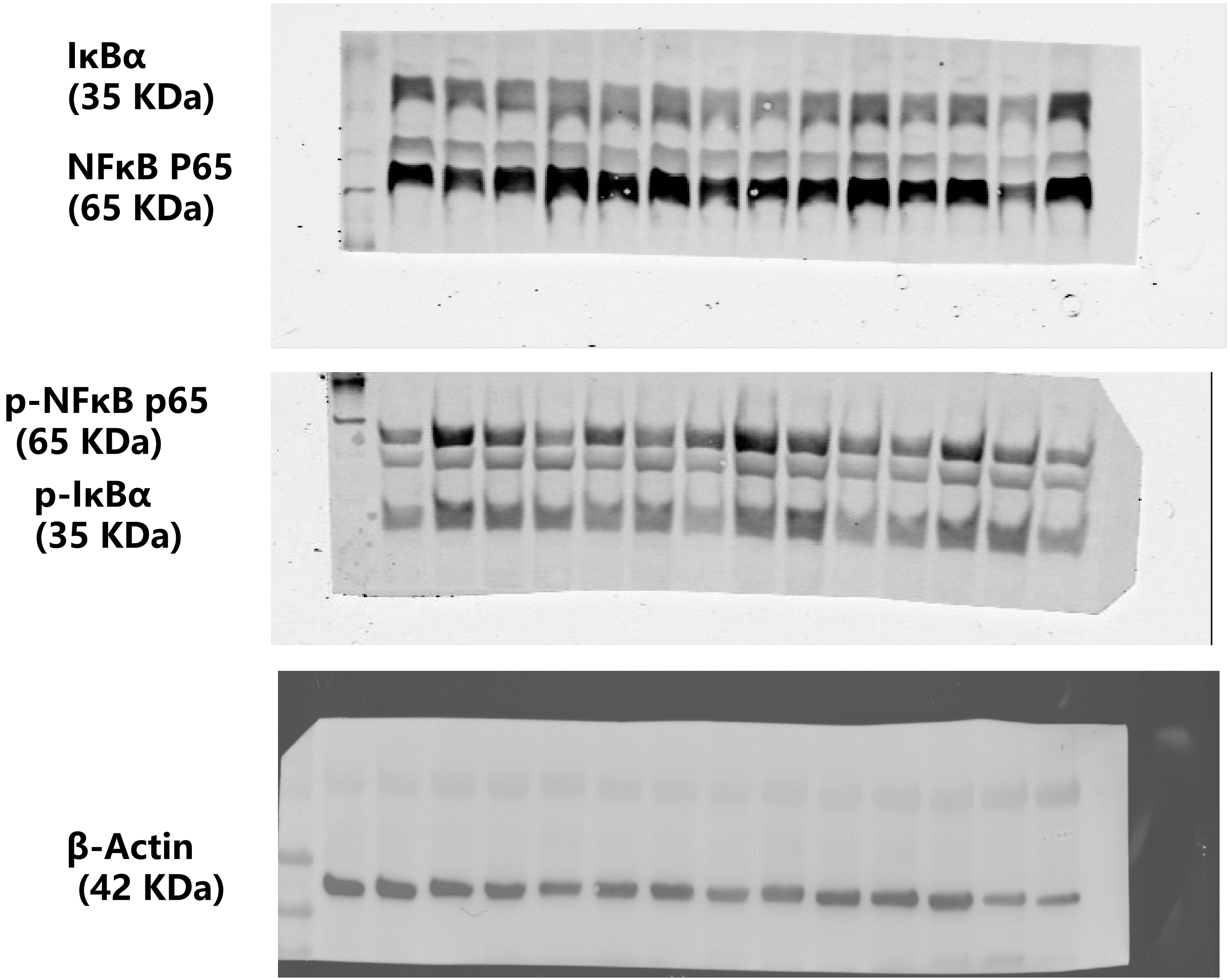

WB replicates original image (TH and β-Actin)

TH(60KDa)

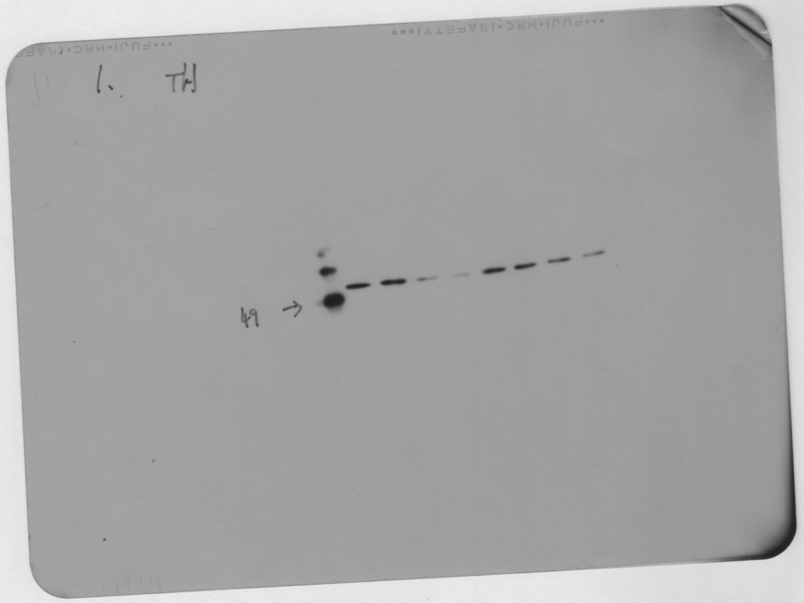

β-Actin
(42KDa)

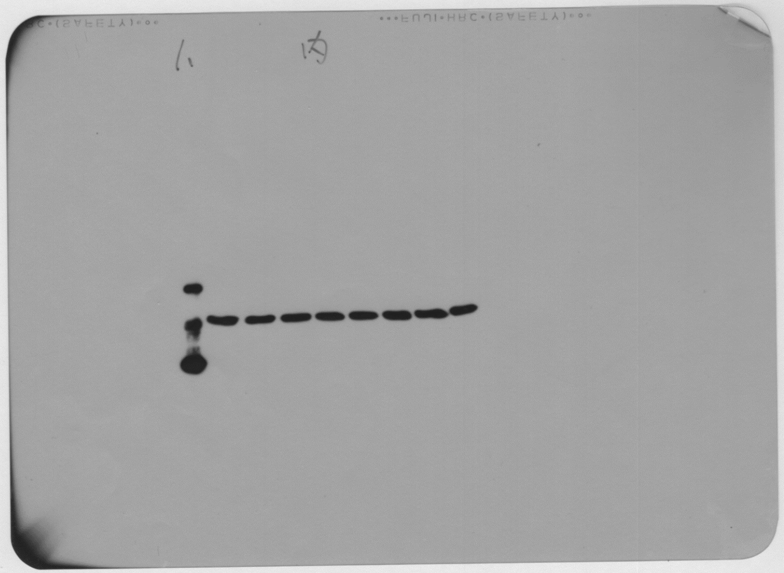

TH(60KDa)

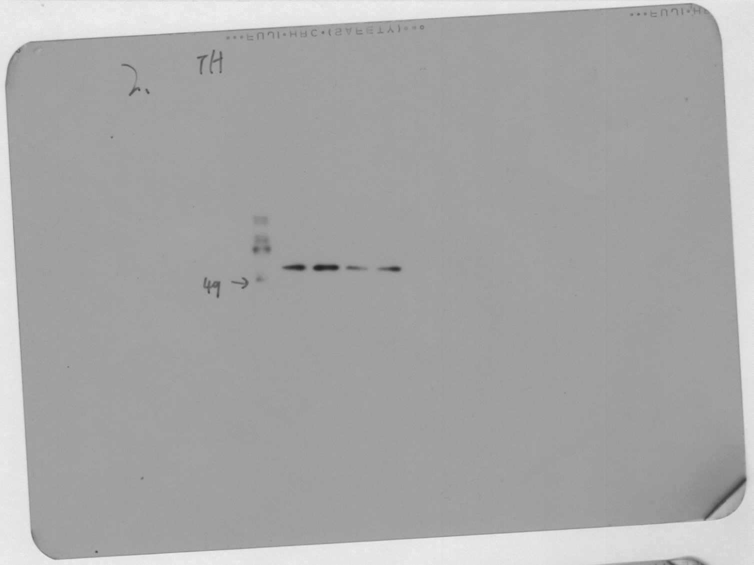

β-Actin
(42KDa)

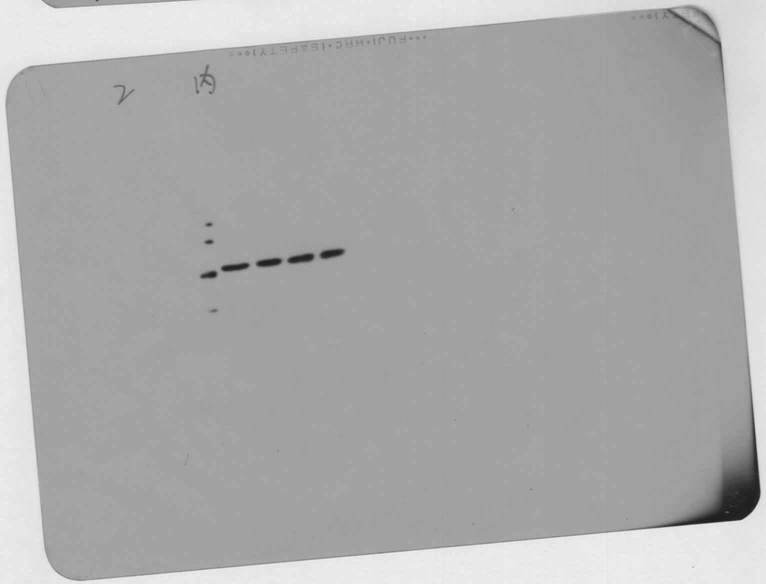

WB replicates original image (p-IκBα ,IκBα and β-Actin)

IκBα
(35 KDa)

p-IκBα
(35 KDa)

β-Actin
(42 KDa)

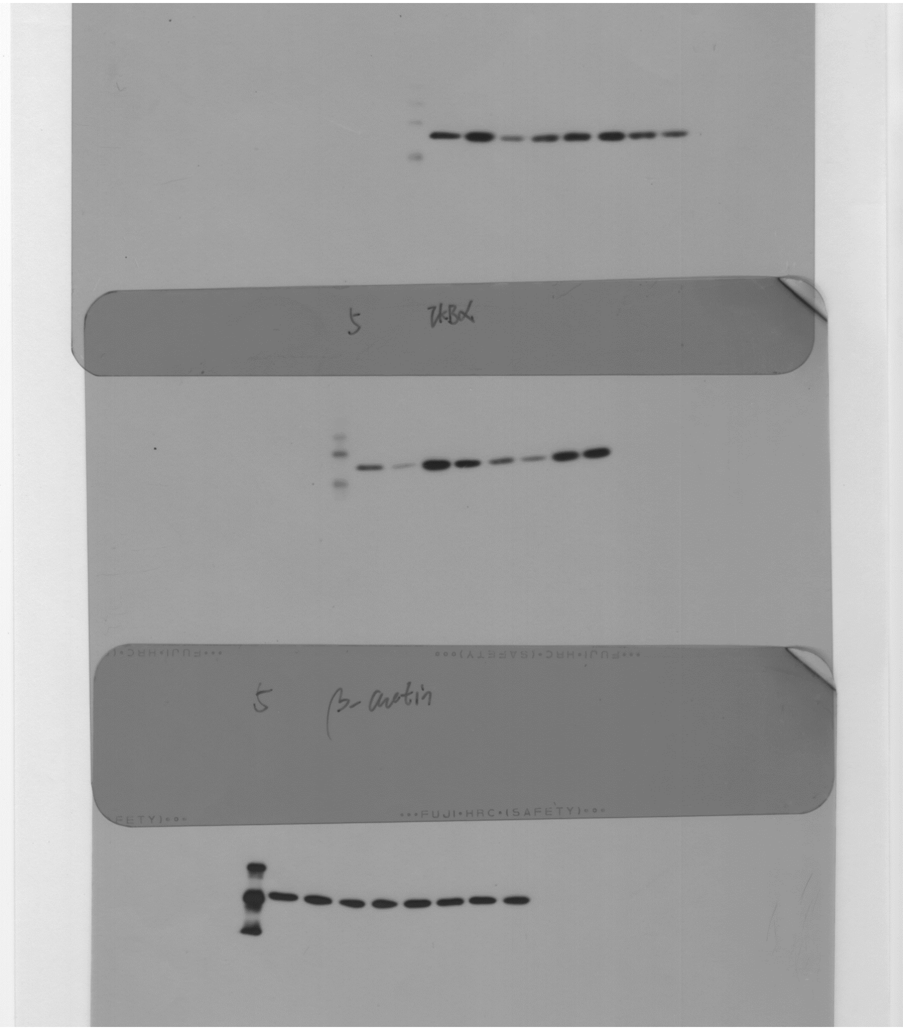

p-IκBα
(35 KDa)

IκBα
(35 KDa)

β-Actin
(42 KDa)

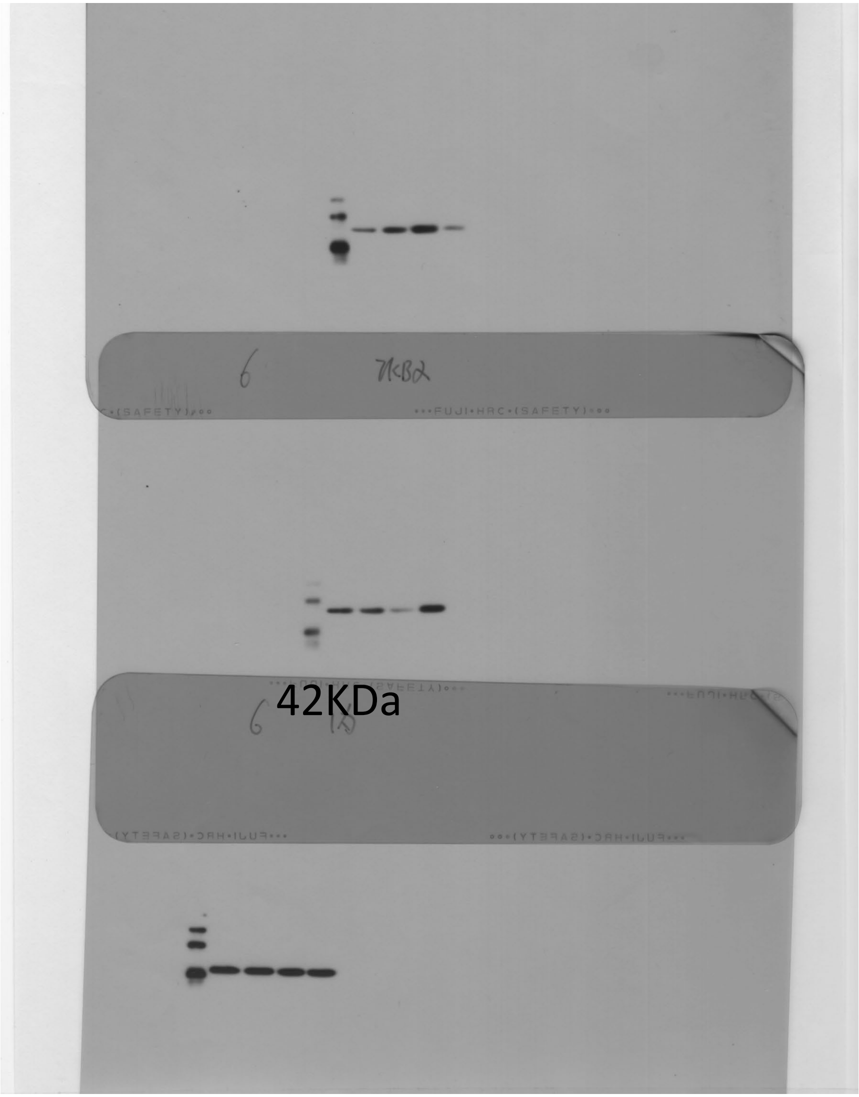

WB replicates original image (p-NFκB p65 ,NFκB p65 and β-Actin)

p-NFκB p65 (65 KDa)

NFκB P65 (65 KDa)

β-Actin (42 KDa)

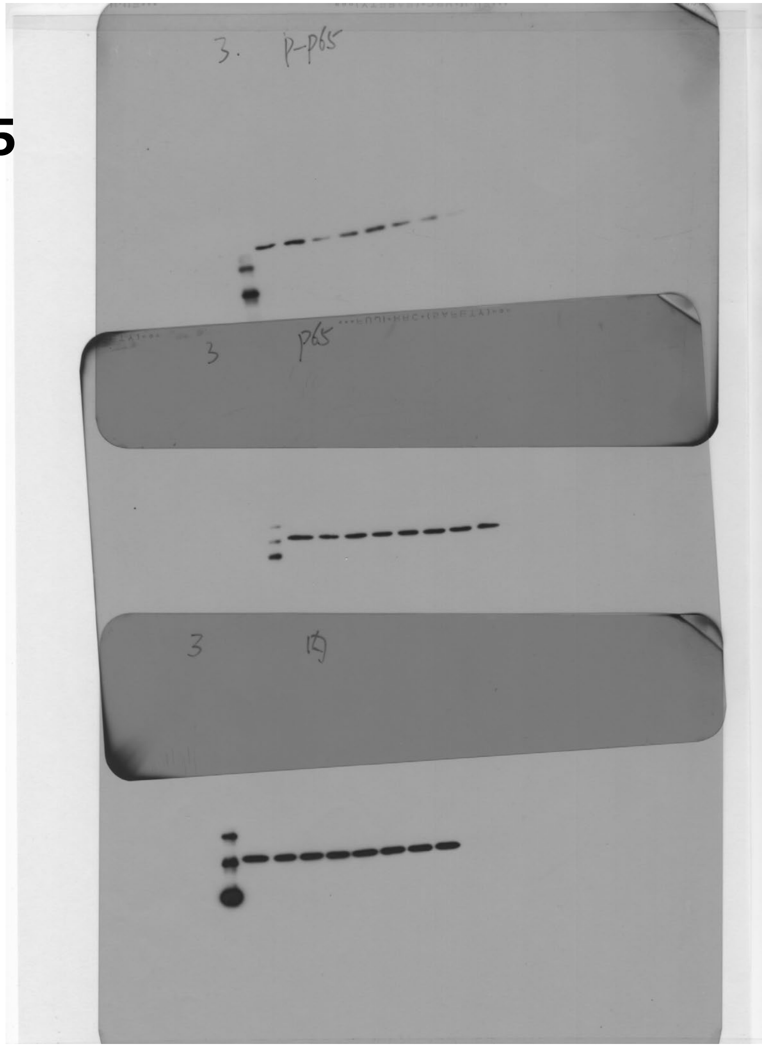

p-NFκB p65 (65 KDa)

NFκB P65 (65 KDa)

β-Actin (42 KDa)

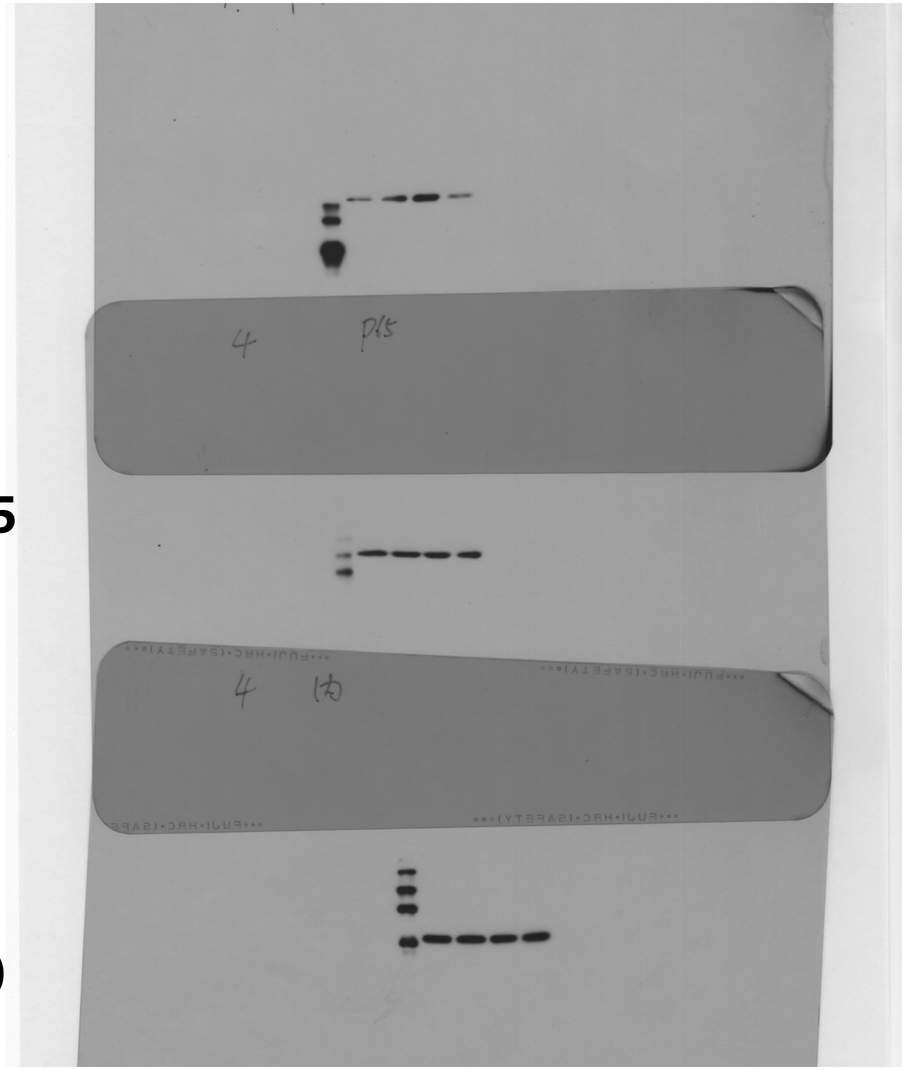

Supplement: Supplementary file 2 — Additional file 2. [file 12864_2022_9073_MOESM2_ESM.pdf]
